# Activation of *CYCD7;1* in the central cell and early endosperm overcomes cell‐cycle arrest in the Arabidopsis female gametophyte, and promotes early endosperm and embryo development

**DOI:** 10.1111/tpj.12957

**Published:** 2015-10-01

**Authors:** Emily Sornay, Céline Forzani, Manuel Forero‐Vargas, Walter Dewitte, James A.H. Murray

**Affiliations:** ^1^ Cardiff School BiosciencesCardiff UniversitySir Martin Evans buildingMuseum AvenueCardiffCF10 3AXWalesUK; ^2^ Institut Jean‐Pierre BourginINRA Centre de Versailles‐GrignonRoute de Saint‐Cyr78026VersaillesCedexFrance; ^3^ Facultad de IngenieriaUniversidad de IbaguéCalle Barrio AmbaláIbagué730002Colombia

**Keywords:** Arabidopsis, D‐type cyclins, seed size, cell cycle, endosperm, central cell

## Abstract

In angiosperms, double fertilization of the egg and central cell of the megagametophyte leads to the development of the embryo and endosperm, respectively. Control of cell cycle progression in the megagametophyte is essential for successful fertilization and development. Central cell‐targeted expression of the D‐type cyclin *CYCD7;1* (*_end_CYCD7;1*) using the imprinted *FWA* promoter overcomes cycle arrest of the central cell in the Arabidopsis female gametophyte in the unfertilized ovule, leading to multinucleate central cells at high frequency. Unlike *FERTILIZATION‐INDEPENDENT SEED* (*fis*) mutants, but similar to lethal *RETINOBLASTOMA‐RELATED* (*rbr*) mutants, no seed coat development is triggered. Unlike the case with loss of *rbr*, post‐fertilization *_end_CYCD7;1* in the endosperm enhances the number of nuclei during syncytial endosperm development and induces the partial abortion of developing seeds, associated with the enhanced size of the surviving seeds. The frequency of lethality was less than the frequency of multinucleate central cells, indicating that these aspects are not causally linked. These larger seeds contain larger embryos composed of more cells of wild‐type size, surrounded by a seed coat composed of more cells. Seedlings arising from these larger seeds displayed faster seedling establishment and early growth. Similarly, two different embryo‐lethal mutants also conferred enlarged seed size in surviving siblings, consistent with seed size increase being a general response to sibling lethality, although the cellular mechanisms were found to be distinct. Our data suggest that tight control of CYCD activity in the central cell and in the developing endosperm is required for optimal seed formation.

## Significance Statement

Controlling cell cycle progression in the female gametophyte is essential for fertilization and development. Here we use targeted activation of a cyclin in the central cell to show that division of the central cell nucleus can be uncoupled from egg cell proliferation. Further phenotypes of these lines show that accurate control of *CYCD* activity is needed for seed development.

## Introduction

Seeds are essential for the dispersal and survival of higher plants, and are central to human nutrition. Seeds consist of three compartments – the mature embryo, the endosperm and the encapsulating seed coat – carrying different balances of maternal and paternal genome (Goldberg *et al*., 1994). They arise from a double fertilization of the megagametophyte (ovule), in which one sperm cell fuses with the egg cell, forming a diploid embryo with equal contributions from each parental genome, whereas the other sperm cell fuses with the diploid central cell to form the triploid endosperm carrying a 2:1 ratio of maternal:paternal genome (Faure *et al*., 2002; Berger, 2008). The seed coat protecting the two zygotic tissues derives from the diploid maternal integument.

The success of seed production depends on the proper formation of the female or megagametophyte prior to fertilization (Drews and Koltunow, 2011) and the coordination of growth of the three seed compartments post‐fertilization (Garcia *et al*., 2005). The megagametophyte, embedded in the maternal integuments, arises from syncytial divisions of the haploid nucleus of the megaspore that produce eight nuclei and gives rise to seven cells, which include the egg cell and the single central cell containing two identical haploid nuclei. In the mature female gametophyte, these two haploid central cell nuclei fuse to form a polar diploid nucleus located towards the micropyle, closest to the egg cell (Drews and Koltunow, 2011). Upon delivery of two sperm cells by the pollen tube, fertilization results from the fusion of the two sperm cell nuclei with the nuclei of the egg cell and central cell of the gametophyte. This triggers cell division of the fertilized egg cell to generate the embryo, whereas mitosis of the central cell produces the initially multinucleate syncitial endosperm. Endosperm development, normally in response to fertilization, triggers ovule integuments to differentiate into the seed coat (Garcia *et al*., 2005), although parthenogenetic seed development in the *fertilization‐independent seed* (*fis*) mutants also triggers seed coat differentiation (Chaudhury *et al*., 1998; Ingouff *et al*., 2006). The development of both the megagametophyte and of the seed is therefore characterized by high mitotic activity (Garcia *et al*., 2005; Ingouff *et al*., 2006; Sabelli and Larkins, 2009).

Cell cycle progression in eukaryotes is controlled by the activity of cyclin‐dependent kinases (CDKs). In complex with their cyclin (CYC) regulatory subunit (Kono *et al*., 2007), CDKs form serine‐threonine protein kinases, the activity of which is further controlled by post‐translational modifications and regulatory interactions. In plants, key controllers of CDK/CYC activity are the ICK/KRP and SIM‐related inhibitors (De Veylder *et al*., 2007). The D‐type cyclins (CYCDs) are represented by 10 genes in seven groups in Arabidopsis, and these, associated with the A‐type CDK (CDKA), are responsible for the transition of cells from G1 into S phase of the mitotic cell cycle through the canonical RETINOBLASTOMA‐RELATED (RBR)‐E2F pathway (Boniotti and Gutierrez, 2001; Gutierrez *et al*., 2002). CDKA/CYCD kinases phosphorylate RBR modulating the RBR interaction with transcription factors, including those involved in cell cycle progression, such as E2Fs (Magyar *et al*., 2012), which promote the transcription of genes required for the S phase (Kuwabara and Gruissem, 2014).

Regulation of the cell cycle is required for the correct development of the megagametophyte and the subsequent seed development of Arabidopsis (Johnston *et al*., 2008, 2010; Collins *et al*., 2012). In Arabidopsis *rbr* loss‐of‐function mutants, the central cell of the mature female gametophyte has supernumerary nuclei, suggesting a failure of cell‐cycle arrest (Ebel *et al*., 2004; Johnston *et al*., 2008, 2010). Furthermore, RBR is required for the correct expression of imprinted genes, the transcriptional activity of which is dependent on the parental origin. During female gametophyte development, *MET1*, encoding a mediator of DNA methylation, is transcriptionally repressed by a complex of RBR and the Arabidopsis homologue of yeast MULTICOPY SUPPRESSOR OF IRA1 (MSI1), allowing the activation of imprinted genes in the maternal germ line (Johnston *et al*., 2008; Jullien *et al*., 2008).

The *CYCD3* subfamily of CYCDs has three genes in Arabidopsis, and forms the CDKA‐CYCD3 kinase that phosphorylates RBR (Boniotti and Gutierrez, 2001). The *cycd3‐1;2;3* triple mutant shows reduced cell proliferation in the shoot (Dewitte *et al*., 2007), and embryo development is delayed and the seed abortion is increased (Collins *et al*., 2012). The same authors showed that transactivation of either *CYCD3;1* or *CYCD7;1* in both embryo and endosperm triggered cell division and conferred often lethal defects on the embryo, especially in the case of *CYCD3;1* overexpression; however, a role for CYCD activity in the megagametophyte has not been shown.

Here we report on the effects of targeted upregulation of the core cell cycle component *CYCD7;1* in non‐embryonic tissues, namely the central cell and endosperm. We show that control of *CYCD* activity is required for cell‐cycle arrest in the central cell, and for the proper formation of the female gametophyte and subsequent seed development. Lethality in a subset of developing seeds leads to increased seed size, which appears to be a general phenomenon because of increased cell proliferation in the embryo. The arising larger seeds show enhanced growth, suggesting that partial seed abortion could be used as a tool for increasing seed size when faster seedling development is required.

## Results

### Central cell‐targeted CYCD7;1 overcomes cell‐cycle arrest in the central cell of female gametophyte

**Figure 1:**
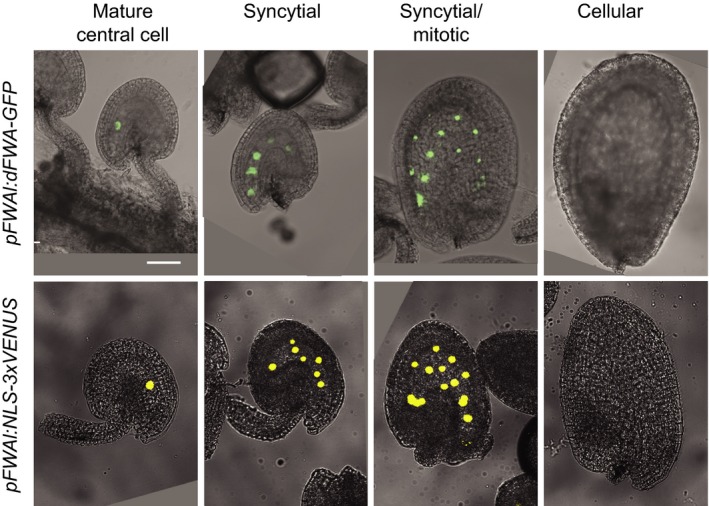
The *FWA cis*‐regulatory sequence drives expression in the central cell and syncytial endosperm. The *FWA* promoter fragment is sufficient to restrict dFWA‐GFP and 3XVENUS expression to the central cell in the mature female gametophyte, and to the syncytial endosperm after fertilization. GFP and 3XVENUS are targeted to the nucleus with the N‐terminal homeodomain fragment of the FWA protein that contains a nuclear‐targeting sequence and an NLS, respectively. Scale bar: 50 μm (shown only in the upper left‐hand panel).

Ectopic expression of *CYCD3;1* and *CYCD7;1* under the control of the non‐specific *RPS5A* promoter in the proliferating tissues conferred developmental defects associated with over‐proliferation (Collins *et al*., 2012). As ectopic expression of *CYCD3;1* led to severe phenotypes by preventing cell division arrest in the suspensor and embryos, we explored the effects of localized *CYCD7;1* activation by using the *FWA* promoter (Kinoshita *et al*., 2004) to target *CYCD7;1* expression specifically to the central cell and endosperm (_*end*_ *CYCD7;1*). Previous work showed that a protein fusion of GFP to the FWA promoter fragment and the N‐terminal homeodomain and nuclear localization signal of the FWA protein (dFWA) is sufficient to target the GFP to the nuclei of the central cell in the female gametophyte, prior to fertilization, and the nuclei of developing endosperm, until cellularization (Kinoshita *et al*., 2004; Figure 1). We therefore tested whether this *FWA* promoter fragment alone is sufficient to target expression to the mature central cell and the developing endosperm. A reporter construct containing a 3.2‐kb fragment of *FWA* promoter driving an NLS‐3XVENUS fusion was examined in Arabidopsis ovules and seeds (Figure 1). Activity from both the dFWA‐GFP protein fusion and the *FWA* promoter reporter was restricted to the mature central cell and developing endosperm. In both transgenic lines, *pFWA:NLS‐3xVENUS* and *pFWA:dFWA‐GFP*, the signal decreased in the early cellularized endosperm, disappeared completely in the cellular endosperm surrounding a heart‐stage embryo (Figure 1) and was absent in a mature male gametophyte. These results corroborate the expression pattern reported by Kinoshita *et al*. (2004), and demonstrate that the promoter fragment alone is sufficient to confer specific expression.

**Figure 2:**
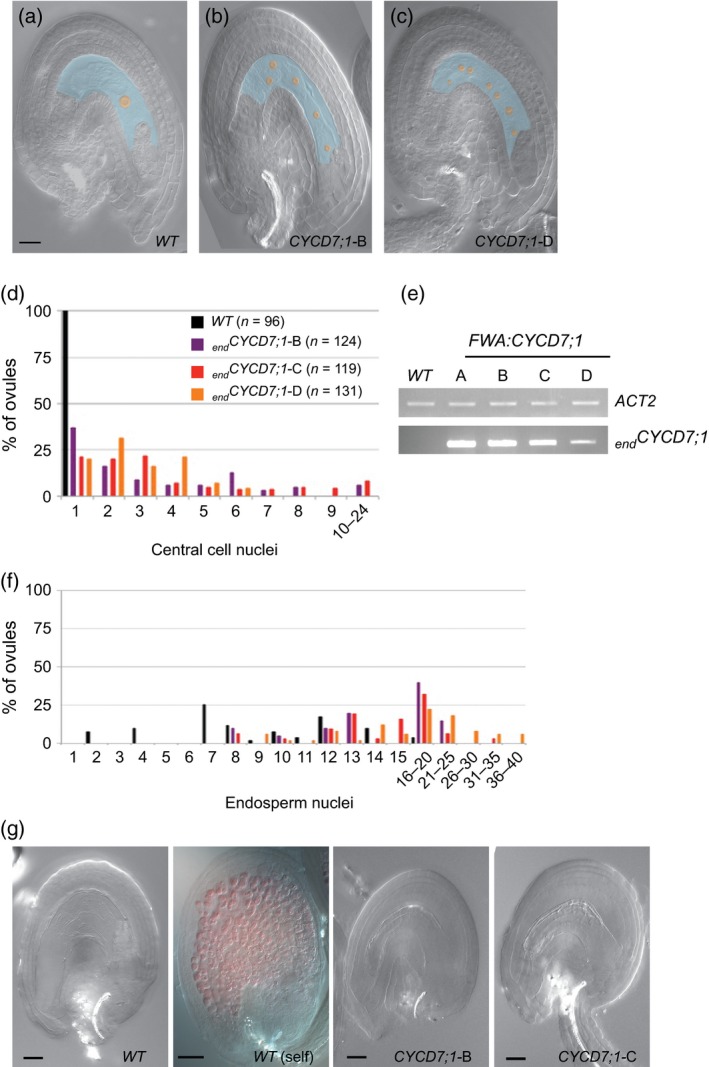
*CYCD7;1* expression in the central cell of the female gametophyte overcomes cell‐cycle arrest. (a–c) Digital interference contrast (DIC) micrographs of unfertilized ovules. Wild‐type (WT) ovules show a single nucleus (orange) in central cell (blue) (a), whereas several nuclei are visible in independent lines of endosperm‐targeted *CYCD7;1* expression (_*end*_ *CYCD7;1*; two representative examples are shown in b and c). Scale bar: 20 μm. (d) Bar chart showing the number of nuclei in the central cell of ovules sampled from WT (top panel) and _*end*_ *CYCD7;1* (bottom panel) emasculated pistils. (e) Estimation of expression level of _*end*_ *CYCD7;1* transcripts in four independent transgenic lines (_*end*_ *CYCD7;1* A–D) by semi‐quantitative RT‐PCR. (f) Number of nuclei in the seed endosperm 24 h after pollination (*n* = 51). (g) Fertilization event triggers the synthesis of proanthocyanidins (PAs) in the maternal integuments marking the acquisition of seed coat fate. PA‐accumulating cells are decorated red after vanillin staining. No PA was detected by this method in _*end*_ *CYCD7;1* ovules. Scale bars: 40 μm.

In unfertilized wild‐type (WT) ovules, the gametophyte central cell contains a single nucleus, located towards the egg apparatus on the micropyle pole of the ovule (Figure 2a). In _*end*_ *CYCD7;1* lines, between 63 and 80% of ovules displayed supernumerary nuclei in the central cell of the unfertilized mature ovule, indicating a failure of cell‐cycle arrest (Figure 2b,c). In lines with higher levels of _*end*_ *CYCD7;1* transactivation (Figure 2e), the number of supernumerary nuclei was found to be higher compared with lines of lower CYCD7;1 expression (Figure 2d), displaying up to around 20 nuclei. In _*end*_ *CYCD7;1* ovules, the supernumerary nuclei are distributed not only towards the micropylar pole but throughout the gametophyte.

The multicellular endosperm in unfertilized ovules of *fie* mutants is a consequence of the fertilization‐independent proliferation of the central cell that occurs together with the acquisition of endosperm fate and the fertilization‐independent differentiation of the seed coat. We therefore examined ovules for evidence of premature seed coat differentiation using vanillin staining (Ingouff *et al*., 2006; Figure 2g), but saw no evidence of this. Hence, although _*end*_ *CYCD7;1* induces central cell nuclear proliferation, the absence of seed coat differentiation suggests that _*end*_ *CYCD7;1* does not trigger autonomous seed development as seen in *fie*. Mutants in the *rbr* gene also show spontaneous nuclear proliferation in female gametophytes in the absence of fertilization, producing up to 20 nuclei (Ingouff *et al*., 2006), very similar to *CYCD7;1* ectopic expression. This confirms that nuclear proliferation does not itself trigger the seed coat differentiation seen in *fie* mutants, and further suggests that CYCD7;1 may be operating by inactivating RBR.

### *CYCD7;1* expression in the endosperm leads to accelerated seed development during the syncytial phase

After fertilization, the triploid endosperm undergoes several rounds of syncytial mitosis (Boisnard‐Lorig *et al*., 2001). Cellularization of the endosperm starts at the micropylar end when the embryo has reached the late globular stage. In _*end*_ *CYCD7;1* lines, the endosperm was observed to develop faster than the WT counterpart. By 24 h after pollination (24 HAP), a high proportion of _*end*_ *CYCD7;1* ovules contain 16–20 nuclei in the endosperm, compared with between seven and nine in the WT ovules (Figure 2f).

**Figure 3:**
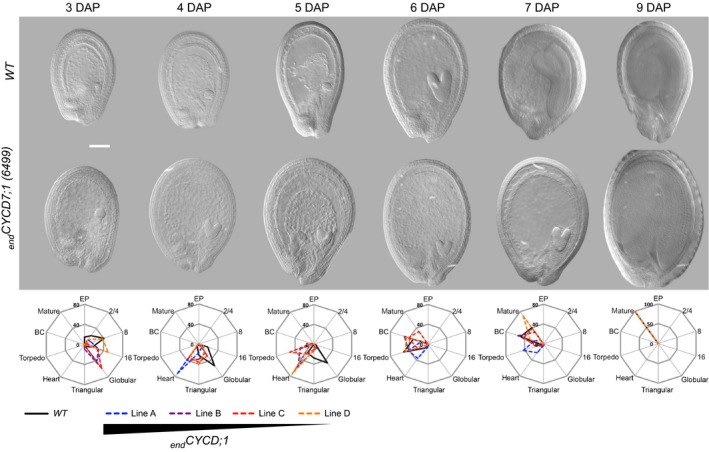
Seed development in wild type (WT) and endosperm‐targeted *CYCD7;1* lines. Upper panel: representative examples of WT and _*end*_ *CYCD7;1*‐A seeds sampled at different time points after pollination (3, 4, 5, 6, 7 and 9 days after pollination, DAP). Scale bar: 50 μm. Middle panel: radar plots depicting the proportion of seed with embryos at different stages at 3, 4, 5, 6, 7 and 9 DAP of WT and _*end*_ *CYCD7;1* lines. For each time point and genotype, the number of seeds scored ranged from 158 to 237. Lower panel: the relative expression of _*end*_ *CYCD7;1* in the different lines is indicated by the triangle.

The rate of embryo development progression was also influenced by _*end*_ *CYCD7;1* (Figure 3). At 3 days after pollination (3 DAP), in WT plants, 20% of embryos reached the 16‐cell stage, 41% reached the 8‐cell stage, 24% reached the 2/4‐cell stage and 15% were in the embryo‐proper stage (immediately after the first division of the zygote). At this point, up to 60% of _*end*_ *CYCD7;1* embryos were already at the globular stage (line C), only 10% were at the 2/4‐cell stage and no embryos were still at the embryo‐proper stage. At 4 DAP, the majority of *WT* embryos (54%) were in the globular stage, only 27% had progressed beyond this point and 19% were still in the 16‐cell stage. _*end*_ *CYCD7;1* embryos were mainly at the triangular and globular stages. At 5 DAP, line A had 72% of embryos at the heart stage and 17% of embryos at the triangular stage. Line D had 28% at the heart stage and 41% at the triangular stage. A high proportion of _*end*_ *CYCD7;1* embryos reached the heart phase faster than in the WT, but the cellular patterning of the heart stage embryo was unaffected (Figure S1).

Finally, from 6 DAP onwards, _*end*_ *CYCD7;1* seed development slowed, with a marked effect on the transition from the heart phase to the torpedo phase. This could be consistent with a reduction of *CYCD7;1* expression in the endosperm at this stage; however, detailed analysis of the different lines showed that from 7 DAP onwards, the line with the strongest level of expression (line A) tends to develop more slowly than the WT, whereas seeds from the line with the lowest expression level are still somewhat further ahead in seed development than WT seeds (Figure 3).

In conclusion, although reaching heart phase faster, _*end*_ *CYCD7;1* embryos stay longer in this phase, with presumably reduced embryo growth at this stage, so that they reach the mature phase at a similar time to the WT. Taking into account the effect of _*end*_ *CYCD7;1* on the population of nuclei during the syncytial phase and *pFWA*‐driven expression in the central cell and syncytial endosperm (see above), it therefore appears that the accelerated endosperm development in _*end*_ *CYCD7;1* lines stimulates the early phases of embryo development, but may subsequently lead to a slower progression to later stages beyond the heart phase.

### Endosperm‐targeted CYCD7;1 produces larger seeds accompanied by lethality

**Figure 4:**
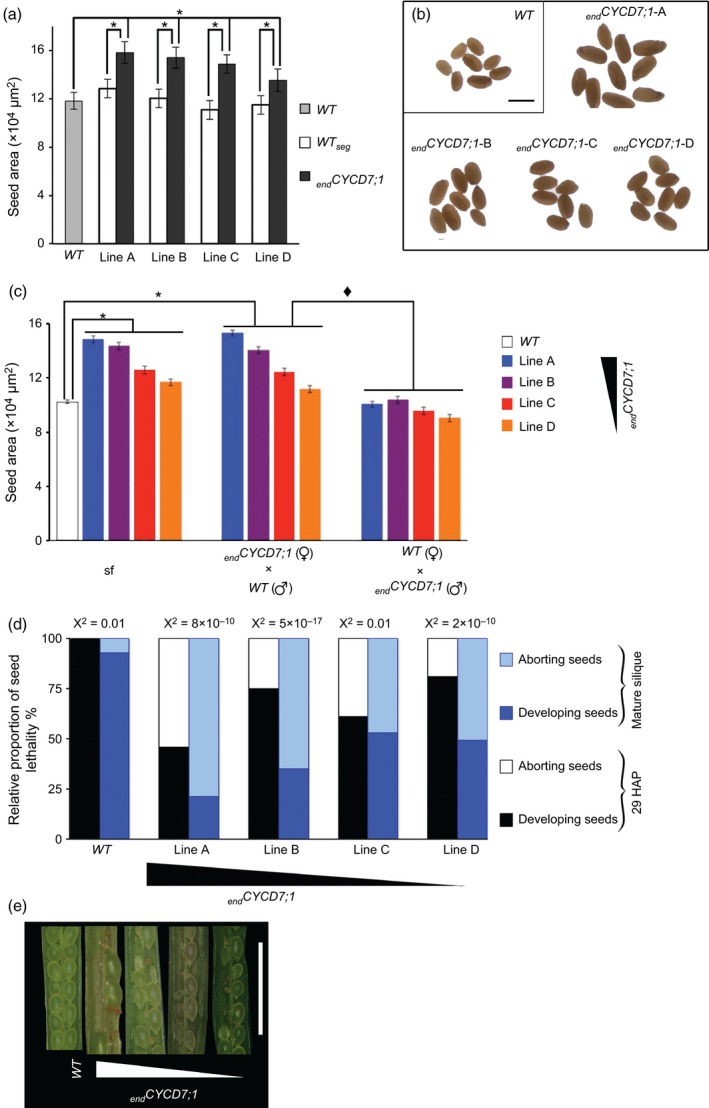
Endosperm‐targeted *CYCD7;1* expression confers a larger seed phenotype that is maternally transmitted. (a) Bar chart presenting average projected areas of mature dry seeds of wild type (WT) and _*end*_ *CYCD7;1* lines. (b) Mature dried seeds from WT *Col‐0* and _*end*_ *CYCD7;1* lines. Scale bar: 500 μm. (c) Comparison of projected area of F_1_ seed generated by crossing WT and _*end*_ *CYCD7;1*. _*end*_ *CYCD7;1* seeds derived by self‐pollination (sf) as well as F_1_ seeds from _*end*_ *CYCD7;1* (female) × *Col‐0* (male) are larger then seeds from *Col‐0* (female) × _*end*_ *CYCD7;1* (male). Diamonds indicate that seed size between the reciprocal crosses was significant, and the asterisk indicates a statistical difference when compared with the WT. The relative expression of _*end*_ *CYCD7;1* in lines A–D is indicated by the triangle. (d) Relative proportion of developing/aborted seeds in siliques from WT and _*end*_ *CYCD7;1* plants (lines A–D). (e) Aborted and developing seeds in WT (first on the top) and _*end*_ *CYCD7;1* siliques (from the second, top to bottom). Scale bar: 2 mm. Error bars represent standard errors on bar charts (*n* = 75).

Several studies have shown that when genes expressed in the different tissues of the ovules and/or developing seeds are misregulated, an effect on the final seed size can be observed (Jofuku *et al*., 1994; Werner *et al*., 2003; Garcia *et al*., 2005; Luo *et al*., 2005; Riefler *et al*., 2006; Schruff *et al*., 2006; Adamski *et al*., 2009). Therefore, we investigated the effect of endosperm‐targeted CYCD7;1 on the size of mature seeds. The size of homozygous _*end*_ *CYCD7;1* seeds was compared with seeds from untransformed *Col‐0* WT plants and from WT segregants derived from the primary transformants, all obtained from plants grown alongside each other under the same conditions (Figure 4a,b). Seed projected area was measured using a custom imagej plug‐in (see Experimental Procedures). A *Col‐0* WT seed had an area of 118 (±18) × 10^3^ μm^2^. WT segregant lines produce seeds with areas ranging from 110 × 10^3^ to 128 × 10^3^ μm^2^, and were no significantly different from the untransformed WT (anova,* P *= 0.08). In contrast, homozygous _*end*_ *CYCD7;1* plants produced significantly larger seeds (55 and 13% larger in lines A and D, respectively; anova,* P *= 7.17 × 10^−19^; Figure 4a). Although untransformed WT and WT segregants display a degree of variation, _*end*_CYCD7;1 OE seeds were consistently larger than their segregating WT counterparts.

The _*end*_ *CYCD7;1* transgenic lines in which *CYCD7;1* expression was under the control of the *FWA* promoter thus produced seeds with an enlarged overall size. The imprinting of the *FWA* promoter region confers female gametophyte‐specific expression because of a lack of methylation of the maternal genome (Kinoshita *et al*., 2004). If the enlarged seed phenotype is caused by _*end*_ *CYCD7;1* expression, we would predict this will only occur from a maternally inherited gene copy. Reciprocal crosses between *Col‐0* WT and _*end*_ *CYCD7;1* lines were performed, as provided that the imprinting is not disrupted, _*end*_ *CYCD7;1* should be expressed only in the female gametophyte. Hence a single maternal copy of _*end*_ *CYCD7;1* should be sufficient to confer the phenotype. WT self‐pollinated negative control seeds had a mean area of 102 × 10^3^ μm^2^ (Figure 4c; Table S1). In the positive controls, where seeds were produced from manual self‐crossing of _*end*_ *CYCD7;1* plants, the seed areas were significantly larger, with average areas of 148 × 10^3^ μm^2^ (line A, 45% increase), 143 × 10^3^ μm^2^ (line B, 40% increase), 125 × 10^3^ μm^2^ (line C, 23% increase), 116 × 10^3^ μm^2^ (line D, 14% increase; anova,* P *= 3.4 × 10^−47^). The seed size increases were similar between manual and natural occurring _*end*_ *CYCD7;1* self‐crosses (Table S1). When _*end*_ *CYCD7;1* pistils were pollinated with WT pollen, seed sizes were similar to the seeds produced by _*end*_ *CYCD7;1* self‐fertilization (anova,* P *= 0.32), and for the four _*end*_ *CYCD7;1* lines, seeds were larger than WT × WT crosses (anova,* P *= 1.44 × 10^−20^). In contrast, when WT pistils were pollinated with _*end*_ *CYCD7;1* pollen, the average seed area did not significantly differ from the average for seeds from WT × WT (*P *= 0.35), and was significantly smaller than the for seeds from _*end*_ *CYCD7;1* self‐fertilized plants (*P *= 3.9 × 10^−4^).

Taken together, these results show that the production of enlarged seed is conferred by the _*end*_ *CYCD7;1* genotype of the female gametophyte.

A negative effect on silique length was observed in the _*end*_ *CYCD7;1* lines, however (Figure S2): WT mature siliques measured on average 1.61 ± 0.17 cm in length, whereas _*end*_ *CYCD7;1* siliques were significantly shorter (line A, 0.86 ± 0.17 cm; line B, 1.20 ± 0.22 cm; line C, 1.36 ± 0.17 cm; line D, 1.28 ± 0.2 cm; anova,* n *= 75, *P* = 3 × 10^−6^). As the siliques were shorter, the total number of healthy and aborted seed in the pods was recorded (Figure S2). In mature WT siliques, 5% of the total number of seeds produced were aborted, whereas in _*end*_ *CYCD7;1* siliques, 80% of seeds were aborted in line A, 65% in line B, and 50% in lines C and D (Figure 4d,e). Hence, in _*end*_ *CYCD7;1* plants, the relative proportion of seed lethality is substantially greater than in the WT (anova,* n* = 75, *P *= 1.33 × 10^−13^).

To understand better the stage at which this lethality arises, we examined siliques 29 h after pollination and found that a significant proportion of developing _*end*_ *CYCD7;1* embryos had aborted (Figure 4d), although this was a lower frequency of abortion than is observed in mature siliques. These siliques showed no aborted embryos (*n* = 128) that had progressed beyond globular phase, suggesting that the early stages of embryo development are particularly susceptible to the effects of _*end*_ *CYCD7;1*.

Given that enlarged seed size was shown to be linked to the maternal genotype, we investigated the parental origin of seed lethality. Reciprocal crosses were performed between WT and _*end*_ *CYCD7;1* plants, and the proportion aborted was recorded in both mature siliques and during early stages (Table 1). A self‐fertilized cross by manual pollination between WT plants produced 49 ± 8 seeds per silique with 7 ± 8 seeds aborting, corresponding to 14% abortion. Manually selfed _e*nd*_ *CYCD7;1* plants produced 38 seeds per silique for line A, 43 seeds per silique for line B, 48 seeds per silique for line C, and 47 seeds per silique for line D. When _*end*_ *CYCD7;1* pistils were manually pollinated with WT pollen, the total number of seeds produced per silique and the proportion of lethality was comparable with _*end*_ *CYCD7;*1 pistils pollinated with _*end*_ *CYCD7;1* pollen for the four _e*nd*_ *CYCD7;1* lines studied (Table 1). On the other hand, a WT pistil pollinated with _*end*_ *CYCD7;1* pollen produced 50 developing seeds with three aborting (for line A), similar to the number of seeds developing and the proportion aborting in the WT pistil pollinated by WT pollen (chi‐squared test, *P *= 0.54). Similar results were found for the three other _*end*_ *CYCD7;1* lines (lines B, C and D; Table 1). From these observations, we conclude that the maternal _*end*_ *CYCD7;1* genotype confers both enlarged seed size and elevated seed lethality.

**Table 1:** Reciprocal crosses between *Col‐0* wild type and _*end*_ *CYCD7;1* lines reveal a maternal origin of seed lethality

♀ × ♂	*n*	Developing seeds	Aborted seeds	Total number of seed	Chi‐squared test Expected values are from the cross with	
					_*end*_ *CYCD7;1* pistil	*Col‐0* pistil
Col × Col	54	42 ± 13	7 ± 8	49 ± 8		
Col × A	30	50 ± 5	3 ± 3	53 ± 4		0.544826851
Col × B	27	44 ± 11	5 ± 8	50 ± 6		0.345231072
Col × C	33	35 ± 10	7 ± 7	43 ± 5		0.236723571
Col × D	30	40 ± 6	3 ± 3	43 ± 4		0.473200418
A × A	32	11 ± 11	26 ± 11	38 ± 9		
A × Col	36	15 ± 2	21 ± 5	36 ± 4	0.12	
B × B	52	8 ± 5	35 ± 8	43 ± 7		
B × Col	31	8 ± 10	34 ± 7	42 ± 6	0.865772375	
C × C	52	27 ± 11	21 ± 9	48 ± 7		
C × Col	30	21 ± 7	20 ± 8	41 ± 8	0.239938989	
D × D	56	22 ± 9	25 ± 9	47 ± 8		
D × Col	30	24 ± 8	22 ± 9	47 ± 4	0.461680188	

### Partial abortion conferred by embryo lethal mutant alleles increased seed size

Whether there is a general relationship between seed size and sibling lethality is unclear. In order to establish whether partial lethality reducing the number of seeds per silique leads to the enlargement of the surviving sibling seeds in other mutant backgrounds, we examined ovule and embryo development and measured seed size in surviving healthy progeny of heterozygous *cdka1;1* and *nclpp2* mutants (Figure S3). *CDKA1;1* encodes the catalytic partner of CYCD and is required for embryo development (Nowack *et al*., 2006). *nclpp2* is a mutant in ATP‐dependent Clp protease proteolytic subunit‐related protein 2, a chloroplastic protein unrelated to cell division control (Kim *et al*., 2009). These mutants were chosen for having a link to cell cycle control in the case of *cdka1;1–1*, and no link in the case of *nclpp2*.

In the unfertilized ovules of the *cdka1;1–1* ^*+/−*^ mutant, no ovules were observed with more than one central cell nucleus in the gametophyte (Figure S3a–c). In the *nclpp2* ^*+/−*^ mutant, 20% of ovules showed an abnormal ovule development. Of the properly developed ovules in *nclpp2* ^*+/−*^, 96% of ovules showed a single central cell nucleus and 4% displayed two or three nuclei in the central cell of the gametophyte. The presence of extra nuclei in the central cell of unfertilized ovules suggests a lack of fusion, uncoordinated cellularization or division in the formation of the female gametophyte and ovules in the *nclpp2* background. These results were in contrast to the _*end*_ *CYCD7;1* ovules with the penetrant supernumerary central cell nucleus phenotype (Figure 2d).

The proliferation of the endosperm post‐fertilization was, on average, delayed in *cdka1;1–1* ^+/−^ and *nclpp2* ^+/−^ progeny when compared with WT and _*end*_ *CYCD7;1* developing seeds (Figure S3d). Although each of these mutant alleles conferred a significant lethality in the mature silique of 45 and 75%, respectively, the timing of the abortion appeared to differ. At 48 h after pollination, 75% of ovules in the *nclpp2* ^*+/−*^ progeny were already degenerating and not progressing in embryogenesis, whereas 25% progressed, albeit with a delay in embryogenesis. In contrast, in the *cdka1;1–1* ^*+/−*^ pistils no ovule degeneration was observed at this point in time, although embryogenesis was delayed (Figure S4b). Nevertheless, despite these substantial differences in the phenotypes leading to lethality, in both cases a substantial increase in average seed size was observed compared with either the progeny of a *Col‐0* WT or the progeny of a WT sibling of the heterozygote mutant (Figure S4a), similar to _*end*_ *CYCD7;1* lines. These observations suggest that, at least under growth‐room conditions, sibling lethality has a strong effect on the size of the remaining seeds, consistent with either the removal of silique space constraints or a strong sink effect of the ovules normally limiting the growth of Arabidopsis *Col‐0* seeds in the silique.

### Larger _*end*_ *CYCD7;1* seeds have more cells in both embryo and seed coat, and display faster seedling establishment

**Figure 5:**
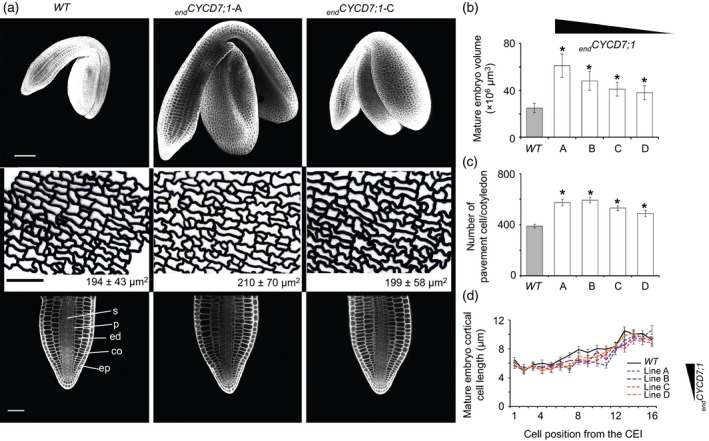
Endosperm‐targeted CYCD7;1 seeds contain elevated numbers of cells in the embryo and seed coat. (a) Phenotype of embryos in mature seeds. Upper row: 3D reconstruction based on confocal *Z*‐stacks of mature embryos. _*end*_ *CYCD7;1* embryos are larger than the WT. Scale bar: 100 μm. Middle row: traces of cell outlines for the abaxial epidermis of the cotyledon. The average pavement cell area is shown in the insert (±SDs). Scale bar: 50 μm. Lower row: longitudinal confocal section of the radicle of mature embryo. Scale bar: 100 μm. Abbreviations: co, cortex; ed, endodermis; ep, epidermis; p, pericycle; s, stele. (b) Quantification of mature embryo volume. (c) Calculated numbers of pavement cells of cotyledons (ratio cotyledon area/epidermal cell area). (d) Quantification of cortical cell length. Error bars show ±SEs. Asterisks indicate a statistical difference in variation of seed size parameters compared with the WT. The relative expression of _*end*_ *CYCD7;1* in the different lines is indicated by the triangle.

In order to understand the larger seed phenotype and the effect of _*end*_ *CYCD7;1* expression, we examined the different seed compartments. A WT embryo had a volume of 25 (±4.7) × 10^6^ μm^3^, whereas _*end*_ *CYCD7;1* embryos had volumes ranging from 61 (±10.1) × 10^6^ μm^3^ (line A) to 38 (±11.3) × 10^6^ μm^3^ (line D), correlated with levels of CYCD7;1 activation (Figure 5a,b). These results show a significant increase in volume, ranging from 50 to 139% (anova, 36 > *n* > 30, *P *= 2.8 × 10^−17^), indicating that the enlarged overall seed size is associated with larger embryos.

No defects in embryonic anatomy were observed (Figure 5a). Confocal examination of cleared mature embryos showed that the _*end*_ *CYCD7;1* radicles have no extra layers, and have a radial pattern similar to that of WT. The length of the first 16 cortical cells, upwards from the cortical–endodermis initial (CEI), was measured. The average length of first cortical cell in a WT root was 6.5 μm. In _*end*_ *CYCD7;1*, cortical cell lengths ranged from 5.5 to 6 μm depending on the line observed (Figure 5d). The sixteenth cortical cell length measured was 9.2 μm in WT, and ranged between 8.8 and 10.6 μm for _*end*_ *CYCD7;1*. In general, _*end*_ *CYCD7;1* cortical cells were slightly smaller than WT equivalents, but a two‐way anova test showed that cortical cell length did not significantly differ between WT and _*end*_ *CYCD7;1* (*n* > 74 cortical files, *P *= 0.23). Overall, the cell length increased up the root, and for both genotypes the cortical cell length is statistically different, depending on the position with respect to the CEI (*n* > 74 cortical files, *P *= 4.6 × 10^−6^).

The cotyledon areas and the average pavement cell areas were also measured in the mature embryo (Figure 5a), making it possible to estimate the number of pavement cells on the surface of the cotyledon. The WT cotyledon surface measured, on average, 75 × 10^3^ μm^2^ (Figures 5a and S5a). _*end*_ *CYCD7;1* measured 120 × 10^3^ μm^2^ (line A), 116 × 10^3^ μm^2^ (line B), 106 × 10^3^ μm^2^ (line C), and 97 × 10^3^ μm^2^ (line D). As the embryo volume increased, the cotyledon area in _*end*_ *CYCD7;1* was significantly enlarged, with a 29–60% increase (anova,* n* > 74, *P *= 1.29 × 10^−5^). The average surface areas of epidermal pavement cells were 194 μm^2^ for the WT and 210, 196, 200 and 199 μm^2^ for _*end*_ *CYCD7;1* lines A, B, C and D, respectively (Figure S5b). A Bonferroni multi‐comparison test revealed that only from _*end*_ *CYCD7;1* line A were the pavement cells significantly larger, by 8% (*n* > 555, *P *= 0.3 × 10^−4^, other comparison *P* > 0.7). The ratio of cotyledon surface:epidermal pavement cell surface showed that the WT area consisted of 388 cells, and that the _*end*_ *CYCD7;1* area consisted area of 572, 589, 529 and 448 cells for lines A, B, C and D, respectively (Figure 5c). WT cotyledons therefore had fewer pavement cells than the _*end*_ *CYCD7;1* lines (anova,* n* > 74, *P *= 7.5 × 10^−20^). Given the small effect on cell size, but the large increase in cell number, we conclude that in _*end*_ *CYCD7;1* lines the major driver towards additional embryo growth is an increase in cell number in the mature embryo and hence cell production during embryo growth.

A similar effect was seen in the _*end*_ *CYCD7;1* seed coat. The size of cells in the outer layer of the outer integument was measured on mature dry seeds. Cells of the outer integuments had on average an area of 778 ± 220 μm^2^ for the WT (Figure S5e). The average cell areas for the _*end*_ *CYCD7;1* lines were 854 ± 256 μm^2^ (line A), 857 ± 261 μm^2^ (line B), 821 ± 285 μm^2^ (line C) and 807 ± 237 μm^2^ (line D), which do not significantly differ from the WT cell area (*n* = 450 for each genotype, anova,* P *= 0.18). The WT seed area was 12.3 × 10^4^ μm^2^, whereas _*end*_ *CYCD7;1* seed areas were significantly larger, with averages of 17.4 × 10^4^ μm^2^ (line A), 17.3 × 10^4^ μm^2^ (line B), 16.2 × 10^4^ μm^2^ (line C) and 14.3 × 10^4^ μm^2^ (line D) (anova,* n* = 250, *P *= 5.9 × 10^−4^). Therefore, the estimated number of cells in the outer integument inferred by the ratio of seed and cell area was 159 for WT. The _*end*_ *CYCD7;1* lines contained significantly more cells, with averages of 204, 203, 197 and 181 for lines A, B, C and D, respectively (anova,* n* = 450, *P *= 6 × 10^−4^). Therefore, the larger _*end*_ *CYCD7;1* seed is linked to more cell proliferation in the various seed compartments rather than increased cell expansion.

Our analysis of surviving siblings in the other embryo‐lethal mutants examined, however, showed that an increase in cell number is not a prerequisite for larger seeds. The surviving seeds derived from heterozygote *cdka1;1–1* ^*+/−*^ and *nclpp2* ^*+/−*^ plants are larger, but on average contain fewer cells in the outer integuments, suggesting that cell expansion is underpinning the increase of seed size in these mutants (Figure S5).

**Figure 6:**
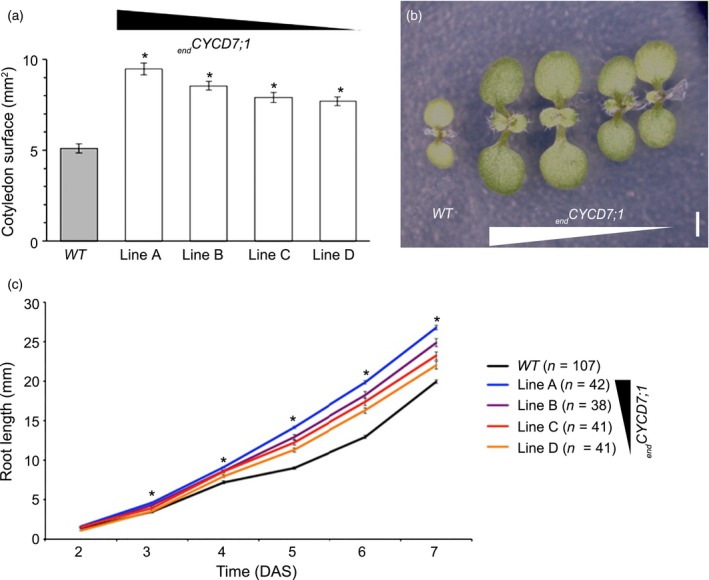
Endosperm‐targeted *CYCD7;1* stimulates seedling establishment. (a) Cotelydon surface areas from 7‐day‐old wild‐type (WT) and _*end*_ *CYCD7;1* seedlings (60 < *n* < 90). (b) Seven‐day old seedlings: from left to right, WT and _*end*_ *CYCD7;1* lines A, B, C and D. Scale bar: 1 mm. (c) Average primary root length in WT and _*end*_ *CYCD7;1* (lines A–D) seedlings 3–7 days after stratification (30 < *n* < 45). The relative expression of _*end*_ *CYCD7;1* in the different lines is indicated by the triangle. Asterisks show statistical difference in root length at each time point compared with the WT.

In order to test whether or not the larger seed sizes of _*end*_ *CYCD7;1* plants would influence the early development of the seedlings, we compared germination rate and seedling development up to 7 days in WT and _*end*_ *CYCD7;1* lines (Figure 6). No major effect on germination rate was recorded, 50% of seeds in all lines germinated between 23 and 26 h, and after 30 h all seeds had germinated (Figure S6). Two days after stratification, primary root length did not differ between *WT* and _*end*_ *CYCD7;1* lines, but from 3 days after stratification the rate of primary root growth of _*end*_ *CYCD7;1* lines was faster (Figure 6C). _*end*_ *CYCD7;1* lines displayed larger cotelydons when measured 7 days after stratification (Figure 6a,b). It thus seems that the larger seeds and embryo size provide an advantage for the early establishment of the seedlings, potentially through the larger embryo and cotyledon size.

## Discussion

The RBR pathway is a key controller of endosperm development in both Arabidopsis (Ebel *et al*., 2004) and *Zea mays* (maize) (Sieberer *et al*., 2009). Ovules carrying the *rbr1* loss‐of‐function mutation in the *RBR* gene megagametophytes are aborted (Ebel *et al*., 2004), and in the absence of fertilization, spontaneous excessive proliferation is observed at the micropylar end that normally develops the egg apparatus and the central cell, producing up to 20 nuclei (Ingouff *et al*., 2006).

Mutants of the *fis* class can initiate parthenogenetic seed development in the absence of fertilization. This is characterized by an autonomous endosperm developing from the central cell, coupled with growth and differentiation of the seed coat (Debeaujon *et al*., 2003; Ingouff *et al*., 2006). This mutant class includes *medea*,* fis2*,* fertilization independent endosperm* (*fie*) and *msi1* (Ohad *et al*., 1996; Chaudhury *et al*., 1998; Chaudhury and Berger, 2001; Kohler *et al*., 2003; Guitton *et al*., 2004); however, unlike the *fis* mutants, growth and differentiation of the seed coat does not occur in *rbr1* (Ingouff *et al*., 2006).

D‐type cyclins can inactivate RBR by hyperphosphorylation (Boniotti and Gutierrez, 2001), and here we show that targeting CYCD7;1 to the central cell phenocopies aspects of the *rbr* mutant phenotype. FWA‐directed expression of *CYCD7;1* expression in the central cell after the last mitosis overcomes cell‐cycle arrest and induces nuclear proliferation in the majority of unfertilized ovules to produce multinucleate ovules at high frequency, but does not affect the formation of the egg apparatus. Although *RBR* expression is detectable in mature ovules only in the central cell, it is expressed throughout megagametophyte development (Ingouff *et al*., 2006). Targeted inactivation of RBR in the central cell therefore allows the effect of overcoming cell‐cycle arrest to be separated from possible effects of RBR on megagametophyte development itself (Ingouff *et al*., 2006). As we only observe a specific effect of CYCD7;1 expression upon the induction of central cell divisions without ovule lethality to the same extent as observed with the loss of function of *rbr1*, we suggest that the lethality in *rbr1* may be related to earlier functions of RBR in megagametophyte development. Although we have no direct evidence that *CYCD7;1* expression specifically directs the phosphorylation of RBR in the central cell, the similarity of this aspect of the phenotype is consistent with this conclusion. Alternatively, CYCD7;1 might indirectly upregulate CDK activity, for example by sequestering and thereby inactivating the CDK kinase inhibitors of the SIM or ICK/KRP family. In any case, our data indicate that proliferation of the central cell nucleus can be uncoupled from the proliferation of the egg cell to some extent without impinging on the viability of the ovule. It is also important to note that the frequency of multinucleate central cell nuclei is much higher (Figure 2d) than the subsequent embryo lethality that we observe (discussed further below), and so the presence of multiple nuclei in the central cell is not in itself sufficient to cause later lethality.

Normally after fertilization the endosperm rapidly triggers sustained cell division and differentiation in the seed coat (Ingouff *et al*., 2006), and this same link between endosperm and seed coat development is seen in *fis* mutants, even in the absence of any parthenogenetic embryo, as in *fis2*; however, the excessive nuclear proliferation in the *rbr1* female gametophyte does not trigger the growth and differentiation of a seed coat, and although the proliferative aspect of the *rbr1* phenotype resulting in supernumerary nuclei is superficially similar to *fis* mutants, they appear to derive from continued proliferation of the central cell lineage, and not from an autonomous seed developmental programme (Ingouff *et al*., 2006). The absence of seed coat development before fertilization in _*end*_ *CYCD7;1* lines, based on the absence of vanillin staining, indicates that the initiation of the proliferation of central cell nuclei by CYCD7;1 is not sufficient to trigger other aspects of seed development, such as seed coat differentiation, as is also observed in *rbr1* mutants.

The use of ectopic _*end*_ *CYCD7;1* expression allows us to examine their effect post‐fertilization, which is not possible in *rbr1* mutants. An increase in nuclear proliferation in the syncytial phase was observed, in line with the canonical role of D‐type cyclins in the promotion of the mitotic cell cycle, presumably as a consequence of continued RBR inactivation. Only progression through the early stages of embryo development was stimulated by _*end*_ *CYCD7;1* in the endosperm, and after reaching heart phase, the development of _*end*_ *CYCD7;1* embryos slowed, so that WT and overexpressor reached maturity around the same time. These results show that increased proliferation within the endosperm strongly promotes early embryo growth. This is consistent with the conclusion that seed size is controlled by the endosperm (Garcia *et al*., 2003, 2005); however, previous analysis has suggested that integument cell proliferation and endosperm growth are largely independent, and cell elongation in the seed coat driven by growth in the endosperm appears to play the major role in the control of seed size (Garcia *et al*., 2005). This led to the suggestion that the maternal‐derived diploid seed coat regulates endosperm growth, which in turn affects the final seed size; however, the targeted expression of *CYCD7;1* only to the endosperm led to increased endosperm nuclear proliferation, associated in the resulting larger seed with an increased number of integument cells. This is consistent with a more dynamic interplay of signals between endosperm and integument than previously suggested, with increased endosperm division triggering increased integument division.

Seed size has been reported to be affected by a range of genetic, epigenetic and environmental factors, particularly internal developmental signals originating from both maternal sporophytic and from post‐fertilization tissues of the endosperm and embryo (Li and Li, 2015). The maternal integuments around the ovule go on to form the seed coat post‐fertilization, and have been proposed to set an upper limit to final seed size acting through the ubiquitin receptor DAR1 (Jofuku *et al*., 1994; Adamski *et al*., 2009; Fang *et al*., 2012; Xia *et al*., 2013). Further maternally acting factors include the transcription factor APETALA 2, and other components of the ubiquitin pathway (Jofuku *et al*., 2005; Li *et al*., 2008; Adamski *et al*., 2009; Ohto *et al*., 2009; Xia *et al*., 2013). After fertilization, several factors have been identified that lead to small seeds through an effect on the regulation of endosperm growth in Arabidopsis (Garcia *et al*., 2003; Kesavan *et al*., 2013; Luo *et al*., 2005; Zhou *et al*., 2009; reviewed Sundaresan, 2005). These include HAIKU 1 (IKU1), IKU2, MINISEED 3 (MINI3) and SHORT HYPOCOTYL UNDER BLUE 1 (SHB1), which function in the same genetic pathway to promote endosperm growth (Garcia *et al*., 2003; Luo *et al*., 2005; Zhou *et al*., 2009). The IKU pathway regulates cytokinin levels in the endosperm through the cytokinin‐degrading enzyme CYTOKININ OXIDASE 2 (*CKX2*), and this is proposed to integrate genetic and epigenetic regulation of endosperm growth (Tanurdzic *et al*., 2008).

Our results showing that lethality in sibling embryos can, by itself, lead to the increased size of surviving seed may require some of these analyses to be reinterpreted. We found that surviving seeds in _*end*_ *CYCD7;1* lines produce larger seeds, as do two other heterozygote mutants that segregate lethal homozygous embryos. The timing of abortion does not seem to be critical to this effect, as _*end*_ *CYCD7;1* and *nclpp2* abort before the globular stage, either without fertilization or in the pre‐globular phases of embryogenesis, whereas the *cdka1;1* mutants abort later. Hence a mutant associated with reduced seed production may lead to the production of larger seeds through a general response of the plant, rather than a specific effect on seed development. Given the fewer but larger cells in the integuments in surviving siblings in lines with *nclpp2* and *cdka1;1* mutations, larger seeds are not necessarily composed of tissues with more cells, but can also arise from promoting cell expansion.

Besides the effects in the developing seed, there thus appears to be a reciprocal relationship between ovule numbers and seed size, suggesting a potential nutrient sink effect on seed size. Indeed, a similar effect was also found in other mutants with a reduction of seed numbers, either by abortion or reduced initiation. For example, an increase in seed size was found in other mutants with a reduced number of seeds in *eod3‐1D* and *ap2* (Jofuku *et al*., 2005; Riefler *et al*., 2006; Ohto *et al*., 2009; Fang *et al*., 2012). Reduced CK signalling resulted in fewer yet larger seeds in the *ahk2‐3‐4* mutants in multiple cytokinin receptors (Riefler *et al*., 2006), and mutants in the cytokinin transcription factors *arr1‐10‐12* also have shorter siliques containing larger seeds (Lee *et al*., 2010). This could well reflect specific cytokinin effects, but may also be the result of altered numbers. Hence, we suggest that although an observed effect on seed size certainly does not exclude specific action of regulatory pathways during seed formation, the effect of sink size as a result of ovule number and/or lethality may provide an interfering influence that must be considered. Our analysis thus suggests that careful analysis is required before concluding that specific mutations lead to seed size increase if there is associated embryo lethality.

We expressed _*end*_ *CYCD7;1* under the *FWA* promoter, which is normally imprinted and only maternally expressed. Consistent with this, we saw lethality and the larger seed phenotype only when _*end*_ *CYCD7;1* was of maternal origin, indicating that the transgene is correctly controlled by imprinting and that the maternal allele is expressed in the developing seed. In this case, the increase in seed size was associated with increased nuclei in the endosperm, but also with elevated cell numbers in both the seed coat and mature embryo. In these seed compartments, which were not exposed to ectopic *CYCD7;1* expression, the relationship between cell growth and cell division was unaltered, resulting in larger seeds containing larger embryos composed of more cells, without an effect on cell size. The reduction of the level of ICK/KRP CDK inhibitors in higher‐order *krp* mutants (Callard *et al*., 1996) also conferred larger seeds with larger embryos; however, in this case the relationship between cell growth and cell size was found to be altered, and embryos were composed of smaller cells, indicating that KRPs mediate cell size within the embryo itself. A similar increase in seed size was observed upon stimulating the CLE8‐WOX8 pathway, not cell‐autonomously involved in coordinating cell proliferation in the embryo and endosperm (Blom *et al.,* 1992), and therefore an attractive hypothesis is that enhanced cell proliferation in the endosperm also stimulates cell proliferation in the embryo.

As the expression of _*end*_ *CYCD7;1* induced partial seed abortion, and partial ovule abortion in embryo‐lethal mutants also increased the size of surviving seeds in our studies, the sink effect potentially provides a confounding influence with respect to overall seed size. We cannot therefore distinguish whether the enlarged seed phenotype is the result of stimulation of endosperm cell division, triggered by ectopic CYCD7;1, or the result of additional resources being available to each developing seed as a consequence of sibling mortality.

In conclusion, ectopic expression of the D‐type cyclin CYCD7;1 in the central cells overcomes cell‐cycle arrest in the female gametophyte and stimulates the syncytial divisions in the endosperm upon fertilization. This negatively affects seed development because a subset of developing seeds aborts, indicating that tight regulation of cell division in the female gametophyte and developing endosperm is required. Nevertheless, the surviving seeds are larger and the resulting seedlings become established more quickly, possibly through the relocation of maternal resources.

## Experimental Procedures

### Plant material and growth conditions

Seeds were surface sterilized with 5 g L^−1^ Chlorifix (sodium dichloroisocyanurate; Bayrol) in 70% ethanol, washed three times with 70% ethanol. Sterilized seeds were dispersed on GM medium [4.4 g L^−1^ MS medium (Sigma‐Aldrich, http://www.sigmaaldrich.com), 1.5% sucrose and 1% agar]. The seeds were stratified (4°C for 48 h) and germinated on GM, then transferred onto soil. Plants were grown at 21°C under a 16‐h light/8‐h dark photoperiod.

*Arabidopsis thaliana* ecotype *Columbia* (*Col‐0*) was used. *pFWA:NLS‐3xVENUS* and *pFWA:CYCD7;1* constructs were introduced using floral‐dipping methods (Clough and Bent, 1998). Primary transformants were selected on GM medium containing kanamycin (50 μg ml^−1^) or d,l‐phosphinothricin (15 μg ml^−1^). The transgenic line carrying pBCH2‐pFWA:dFWA‐GFP was used (Kinoshita *et al*., 2004). The embryo‐lethal mutants used were were *cdka1;1+/−* (SALK_106809; Nowack *et al*., 2006) and *nclpp2* (SALK_016774; Rudella *et al*., 2006) in the *Col‐0 A. thaliana* background.

### Plasmids and construct

A 3.2‐kb region upstream of the *FWA* (*At4g25530*) translational start was amplified by PCR from genomic Arabidopsis *Col‐0* DNA and subcloned *Hind*III*‐BamH*I in the pBluescript SK‐II (*pBSC_pFWA*) (Addgene, http://www.addgene.org). The *CYCD7;1* coding sequence was amplified by PCR from cDNA derived from *Col‐0* seedlings. The *CYCD7;1* coding sequence was then inserted in *BamH*I‐*Sac*I in the *pBSC_pFWA*. The *pFWA:CYCD7;1* cassette was excised from *Hind*III‐*Sac*I and inserted in the plant binary vector *pGPTV‐bar* (Becker *et al*., 1992).

To construct the transcriptional reporter *pFWA:3xVENUS*, the *FWA* promoter fragment and 3xVENUS fragment were excised from *pBSC_FWA* (*Hind*III‐*BamH*I) and *pCUC2:3xVENUS* (*BamH*III‐*Not*I), respectively (Heisler *et al*., 2005). The two fragments were inserted in the plant binary vector pGreenI‐0029 between *Hind*III and *Not*I sites (Rudella *et al*., 2006).

### RT‐PCR

Total RNA was extracted from siliques emerging from flowers using the RNeasy Plant mini Kit (Qiagen, http://www.qiagen.com). Total RNA (0.5 μg) was used to perform a OneStep RT‐PCR (Qiagen). *ACTIN2* transcripts were used as a reference. Primer sequences are available on request, and the intensity of the amplicons was compared on agarose gels.

### Whole‐mount staining for the detection of seed coat development

Pistils were emasculated and left for 3 days before sampling. Emasculated pistils (1.5 days old) from the WT were pollinated and left for 1.5 days before sampling.

The ovules or seeds from *WT* and _*end*_ *CYCD7;1* were dissected from pistils, and siliques were incubated in an acidic solution (6 N HCl) of 1% (w/v) vanillin (Sigma‐Aldrich) at room temperature (20°C) for 20 min and rinsed in H_2_O prior to differential interference contrast (DIC) microscopy. Vanillin reacts with the proanthocyanidins synthesized by the seed coat of the fertilized ovules, producing a red reaction product (Debeaujon *et al*., 2003).

### Microscopy of ovule and seeds

For examination with DIC optics, seeds and ovules were cleared and mounted in chloral hydrate/30% glycerol (1:1; 30 ml H_2_O, 80 g chloral hydrate; C8383, Sigma‐Aldrich). Cleared samples were observed using a Zeiss Axio Imager M1 microscope equipped with DIC optics (http://www.zeiss.com).

Seed developmental progression was recorded after hand pollination of emasculated flowers. For each time point (3, 4, 5, 6, 7 and 9 days after pollination, DAP), approximately 230 seeds were scored for each line. The percentage of seeds at each embryo stage was recorded (West and Harada, 1993).

Seed abortion frequency was also scored in hand‐pollinated siliques.

Mature dry seeds were photographed under a Leica microscope (MZ16F) using a LeicaFire Cam digital camera (http://www.leica.com).

For the detection of fluorescence by confocal microscopy (Zeiss LSM‐710), the following light‐path settings were used: YFP‐VENUS was excited with 514 nm and emission was recorded by a 519–621 nm bandpass filter; and for propidium iodide, excitation was set at 543 nm, and emission recorded through a 493–572 nm bandpass filter).

### Cell wall staining of mature embryos and seeds

Dry seeds were imbibed for 2 days in the dark at 4°C. Mature embryos were extracted from the seed coat and fixed with 50% ethanol/10% acetic acid for 24 h, whereas dried mature seeds were fixed directly with 50% ethanol/10% acetic acid for 24 h (Forzani *et al*., 2014). Embryos or seeds were rinsed with water and incubated in 1% periodic acid for 40 min at room temperature. Embryos and seeds were rinsed and stained with Schiff reagent (100 mm sodium metabisulphite, 0.15 N HCl, propidium iodide 100 μg ml^−1^) for 1 h at room temperature. Embryos and seeds were rinsed with water and cleared with a chloral hydrate solution. Embryos and seeds were examined with a confocal laser microscope (Zeiss LSM 710).

### Seed and embryo size measurements

Seed and embryo *Z*‐stack images were processed using ImageJ (Schneider *et al*., 2012). Projective areas of mature dry seeds were generated using Seed Measurer, a purpose‐written plug‐in. Embryonic volume was determined with an imagej plug‐in, embryo 3d, that reconstitutes the 3D structure of the embryo from confocal *Z*‐stacks and calculates the volume.

Statistical analysis using one‐ and two‐way anova and chi‐squared test were performed with the statistics package pasw 16.0 (SPSS Inc., now IBM, http://www.ibm.com). Differences between means for different populations were assumed to be significant when *P* > 0.05.

## Supporting information


**Figure S1.** Heart‐stage embryos in WT and _*end*_ *CYCD7;1* lines.

**Figure S2.** CYCD7;1 expression under the activity of *FWA* induces an increase of seed abortion.

**Figure S3.** Developmental characterization of *cdka1;1–1* ^*+/−*^ and *nclpp2* ^*+/−*^ mutant‐derived ovules and seeds.

**Figure S4.** Characteristics of mutant and _*end*_ *CYCD7;1* seeds.

**Figure S5.** Features of enlarged _*end*_ *CYCD7;1* mature seeds.

**Figure S6.** Germination of seeds derived from _*end*_ *CYCD7;1*, WT and *nclpp2* ^+/−^.

**Table S1.** Reciprocal crosses between *Col‐0* WT and _*end*_ *CYCD7;1* lines reveal a maternal origin of seed size increase (m, manual; sf, self‐pollinated).

## References

[tpj12957-bib-0001] Adamski, N.M. , Anastasiou, E. , Eriksson, S. , O'Neill, C.M. and Lenhard, M. (2009) Local maternal control of seed size by *KLUH/CYP78A5*‐dependent growth signaling. Proc. Natl Acad. Sci. 106, 20115–20120. 19892740 10.1073/pnas.0907024106 PMC2785301

[tpj12957-bib-0002] Becker, D. , Kemper, E. , Schell, J. and Masterson, R. (1992) New plant binary vectors with selectable markers located proximal to the left T‐DNA border. Plant Mol. Biol. 20, 1195–1197. 1463855 10.1007/BF00028908

[tpj12957-bib-0003] Berger, F. (2008) Double‐fertilization, from myths to reality. Sex. Plant Reprod. 21, 3–5.

[tpj12957-bib-0501] Blom, T.J. , Kreis, W. , van Iren, F. and Libbenga, K.R. (1992) A non‐invasive method for the routine‐estimation of fresh weight of cells grown in batch suspension cultures. Plant Cell Rep., 11, 146–149. 24213548 10.1007/BF00232168

[tpj12957-bib-0004] Boisnard‐Lorig, C. , Colon‐Carmona, A. , Bauch, M. , Hodge, S. , Doerner, P. , Bancharel, E. , Dumas, C. , Haseloff, J. and Berger, F. (2001) Dynamic analyses of the expression of the HISTONE:YFP fusion protein in arabidopsis show that syncytial endosperm is divided in mitotic domains. Plant Cell, 13, 495–509. 11251092 10.1105/tpc.13.3.495 PMC135513

[tpj12957-bib-0005] Boniotti, M.B. and Gutierrez, C. (2001) A cell‐cycle‐regulated kinase activity phosphorylates plant retinoblastoma protein and contains, in Arabidopsis, a CDKA/cyclin D complex. Plant J. 28, 341–350. 11722776 10.1046/j.1365-313x.2001.01160.x

[tpj12957-bib-0006] Callard, D. , Axelos, M. and Mazzolini, L. (1996) Novel molecular markers for late phases of the growth cycle of *Arabidopsis thaliana* cell‐suspension cultures are expressed during organ senescence. Plant Physiol. 112, 705–715. 8883383 10.1104/pp.112.2.705 PMC157995

[tpj12957-bib-0007] Chaudhury, A.M. and Berger, F. (2001) Maternal control of seed development. Semin. Cell Dev. Biol. 12, 381–386. 11535046 10.1006/scdb.2001.0267

[tpj12957-bib-0008] Chaudhury, A.M. , Craig, S. , Dennis, E. and Peacock, W. (1998) Ovule and embryo development, apomixis and fertilization. Curr. Opin. Plant Biol. 1, 26–31. 10066555 10.1016/s1369-5266(98)80123-4

[tpj12957-bib-0009] Clough, S.J. and Bent, A.F. (1998) Floral dip: a simplified method for Agrobacterium‐mediated transformation of *Arabidopsis thaliana* . Plant J. 16, 735–743. 10069079 10.1046/j.1365-313x.1998.00343.x

[tpj12957-bib-0010] Collins, C. , Dewitte, W. and Murray, J.A. (2012) D‐type cyclins control cell division and developmental rate during Arabidopsis seed development. J. Exp. Bot. 63, 3571–3586. 22412186 10.1093/jxb/ers015 PMC3388828

[tpj12957-bib-0011] De Veylder, L. , Beeckman, T. and Inze, D. (2007) The ins and outs of the plant cell cycle. Nat. Rev. 8, 655–665. 10.1038/nrm2227 17643126

[tpj12957-bib-0012] Debeaujon, I. , Nesi, N. , Perez, P. , Devic, M. , Grandjean, O. , Caboche, M. and Lepiniec, L. (2003) Proanthocyanidin‐accumulating cells in arabidopsis testa: regulation of differentiation and role in seed development. Plant Cell, 15, 2514–2531. 14555692 10.1105/tpc.014043 PMC280558

[tpj12957-bib-0013] Dewitte, W. , Scofield, S. , Alcasabas, A.A. ***et al.*** (2007) Arabidopsis CYCD3 D‐type cyclins link cell proliferation and endocycles and are rate‐limiting for cytokinin responses. Proc. Natl Acad. Sci. 104, 14537–14542. 17726100 10.1073/pnas.0704166104 PMC1964848

[tpj12957-bib-0014] Drews, G.N. and Koltunow, A.M. (2011) The female gametophyte. Arabidopsis Book, 9, e0155. 22303279 10.1199/tab.0155 PMC3268550

[tpj12957-bib-0015] Ebel, C. , Mariconti, L. and Gruissem, W. (2004) Plant retinoblastoma homologues control nuclear proliferation in the female gametophyte. Nature, 429, 776–780. 15201912 10.1038/nature02637

[tpj12957-bib-0016] Fang, W. , Wang, Z. , Cui, R. , Li, J. and Li, Y. (2012) Maternal control of seed size by *EOD3/CYP78A6* in *Arabidopsis thaliana* . Plant J. 70, 929–939. 22251317 10.1111/j.1365-313X.2012.04907.x

[tpj12957-bib-0017] Faure, J.E. , Rotman, N. , Fortune, P. and Dumas, C. (2002) Fertilization in *Arabidopsis thaliana* wild type: developmental stages and time course. Plant J. 30, 481–488. 12028577 10.1046/j.1365-313x.2002.01305.x

[tpj12957-bib-0018] Forzani, C. , Aichinger, E. , Sornay, E. , Willemsen, V. , Laux, T. , Dewitte, W. and Murray, J.A. (2014) WOX5 suppresses *CYCLIN D* activity to establish quiescence at the center of the root stem cell niche. Curr. Biol. 24, 1939–1944. 25127220 10.1016/j.cub.2014.07.019 PMC4148176

[tpj12957-bib-0019] Garcia, D. , Saingery, V. , Chambrier, P. , Mayer, U. , Jurgens, G. and Berger, F. (2003) Arabidopsis *haiku* mutants reveal new controls of seed size by endosperm. Plant Physiol. 131, 1661–1670. 12692325 10.1104/pp.102.018762 PMC166922

[tpj12957-bib-0020] Garcia, D. , Fitz Gerald, J.N. and Berger, F. (2005) Maternal control of integument cell elongation and zygotic control of endosperm growth are coordinated to determine seed size in Arabidopsis. Plant Cell, 17, 52–60. 15598800 10.1105/tpc.104.027136 PMC544489

[tpj12957-bib-0021] Goldberg, R.B. , de Paiva, G. and Yadegari, R. (1994) Plant embryogenesis: zygote to seed. Science, 266, 605–614. 17793455 10.1126/science.266.5185.605

[tpj12957-bib-0022] Guitton, A.E. , Page, D.R. , Chambrier, P. , Lionnet, C. , Faure, J.E. , Grossniklaus, U. and Berger, F. (2004) Identification of new members of Fertilisation Independent Seed Polycomb Group pathway involved in the control of seed development in *Arabidopsis thaliana* . Development, 131, 2971–2981. 15151989 10.1242/dev.01168

[tpj12957-bib-0023] Gutierrez, C. , Ramirez‐Parra, E. , Castellano, M.M. and del Pozo, J.C. (2002) G(1) to S transition: more than a cell cycle engine switch. Curr. Opin. Plant Biol. 5, 480–486. 12393009 10.1016/s1369-5266(02)00301-1

[tpj12957-bib-0024] Heisler, M.G. , Ohno, C. , Das, P. , Sieber, P. , Reddy, G.V. , Long, J.A. and Meyerowitz, E.M. (2005) Patterns of auxin transport and gene expression during primordium development revealed by live imaging of the Arabidopsis inflorescence meristem. Curr. Biol. 15, 1899–1911. 16271866 10.1016/j.cub.2005.09.052

[tpj12957-bib-0025] Ingouff, M. , Jullien, P.E. and Berger, F. (2006) The female gametophyte and the endosperm control cell proliferation and differentiation of the seed coat in Arabidopsis. Plant Cell, 18, 3491–3501. 17172356 10.1105/tpc.106.047266 PMC1785409

[tpj12957-bib-0026] Jofuku, K.D. , den Boer, B.G. , Van Montagu, M. and Okamuro, J.K. (1994) Control of Arabidopsis flower and seed development by the homeotic gene *APETALA2* . Plant Cell, 6, 1211–1225. 7919989 10.1105/tpc.6.9.1211 PMC160514

[tpj12957-bib-0027] Jofuku, K.D. , Omidyar, P.K. , Gee, Z. and Okamuro, J.K. (2005) Control of seed mass and seed yield by the floral homeotic gene *APETALA2* . Proc. Natl Acad. Sci. 102, 3117–3122. 15708974 10.1073/pnas.0409893102 PMC549499

[tpj12957-bib-0028] Johnston, A.J. , Matveeva, E. , Kirioukhova, O. , Grossniklaus, U. and Gruissem, W. (2008) A dynamic reciprocal *RBR*‐PRC2 regulatory circuit controls arabidopsis gametophyte development. Curr. Biol. 18, 1680–1686. 18976913 10.1016/j.cub.2008.09.026

[tpj12957-bib-0029] Johnston, A.J. , Kirioukhova, O. , Barrell, P.J. , Rutten, T. , Moore, J.M. , Baskar, R. , Grossniklaus, U. and Gruissem, W. (2010) Dosage‐sensitive function of *RETINOBLASTOMA RELATED* and convergent epigenetic control are required during the arabidopsis life cycle. PLoS Genet. 6, e1000988. 20585548 10.1371/journal.pgen.1000988 PMC2887464

[tpj12957-bib-0030] Jullien, P.E. , Mosquna, A. , Ingouff, M. , Sakata, T. , Ohad, N. and Berger, F. (2008) Retinoblastoma and its binding partner MSI1 control imprinting in Arabidopsis. PLoS Biol. 6, e194. 18700816 10.1371/journal.pbio.0060194 PMC2504488

[tpj12957-bib-0031] Kesavan, M. , Song, J.T. and Seo, H.S. (2013) Seed size: a priority trait in cereal crops. Physiol. Plant. 147, 113–120. 22680622 10.1111/j.1399-3054.2012.01664.x

[tpj12957-bib-0032] Kim, J. , Rudella, A. , Ramirez Rodriguez, V. , Zybailov, B. , Olinares, P.D. and van Wijk, K.J. (2009) Subunits of the plastid ClpPR protease complex have differential contributions to embryogenesis, plastid biogenesis, and plant development in Arabidopsis. Plant Cell, 21, 1669–1692. 19525416 10.1105/tpc.108.063784 PMC2714938

[tpj12957-bib-0033] Kinoshita, T. , Miura, A. , Choi, Y. , Kinoshita, Y. , Cao, X. , Jacobsen, S.E. , Fischer, R.L. and Kakutani, T. (2004) One‐way control of *FWA i*mprinting in Arabidopsis endosperm by DNA methylation. Science, 303, 521–523. 14631047 10.1126/science.1089835

[tpj12957-bib-0034] Kohler, C. , Hennig, L. , Bouveret, R. , Gheyselinck, J. , Grossniklaus, U. and Gruissem, W. (2003) Arabidopsis MSI1 is a component of the MEA/FIE *Polycomb* group complex and required for seed development. EMBO J. 22, 4804–4814. 12970192 10.1093/emboj/cdg444 PMC212713

[tpj12957-bib-0035] Kono, A. , Umeda‐Hara, C. , Adachi, S. , Nagata, N. , Konomi, M. , Nakagawa, T. , Uchimiya, H. and Umeda, M. (2007) The Arabidopsis D‐type cyclin CYCD4 controls cell division in the stomatal lineage of the hypocotyl epidermis. Plant Cell, 19, 1265–1277. 17449809 10.1105/tpc.106.046763 PMC1913761

[tpj12957-bib-0036] Kuwabara, A. and Gruissem, W. (2014) Arabidopsis Retinoblastoma‐related and Polycomb group proteins: cooperation during plant cell differentiation and development. J. Exp. Bot. 65, 2667–2676. 24638900 10.1093/jxb/eru069

[tpj12957-bib-0037] Lee, T.J. , Pascuzzi, P.E. , Settlage, S.B. ***et al.*** (2010) *Arabidopsis thaliana* chromosome 4 replicates in two phases that correlate with chromatin state. PLoS Genet. 6, e1000982. 20548960 10.1371/journal.pgen.1000982 PMC2883604

[tpj12957-bib-0038] Li, N. and Li, Y. (2015) Maternal control of seed size in plants. J. Exp. Bot. 66, 1087–1097. 25609830 10.1093/jxb/eru549

[tpj12957-bib-0039] Li, Y. , Zheng, L. , Corke, F. , Smith, C. and Bevan, M.W. (2008) Control of final seed and organ size by the *DA1* gene family in *Arabidopsis thaliana* . Genes Dev. 22, 1331–1336. 18483219 10.1101/gad.463608 PMC2377187

[tpj12957-bib-0040] Luo, M. , Dennis, E.S. , Berger, F. , Peacock, W.J. and Chaudhury, A. (2005) *MINISEED3* (*MINI3*), a WRKY family gene, and *HAIKU2* (*IKU2*), a leucine‐rich repeat (*LRR*) *KINASE* gene, are regulators of seed size in Arabidopsis. Proc. Natl Acad. Sci. 102, 17531–17536. 16293693 10.1073/pnas.0508418102 PMC1297679

[tpj12957-bib-0041] Magyar, Z. , Horvath, B. , Khan, S. , Mohammed, B. , Henriques, R. , De Veylder, L. , Bako, L. , Scheres, B. and Bogre, L. (2012) Arabidopsis E2FA stimulates proliferation and endocycle separately through RBR‐bound and RBR‐free complexes. EMBO J. 31, 1480–1493. 22307083 10.1038/emboj.2012.13 PMC3321179

[tpj12957-bib-0042] Nowack, M.K. , Grini, P.E. , Jakoby, M.J. , Lafos, M. , Koncz, C. and Schnittger, A. (2006) A positive signal from the fertilization of the egg cell sets off endosperm proliferation in angiosperm embryogenesis. Nat. Genet. 38, 63–67. 16311592 10.1038/ng1694

[tpj12957-bib-0043] Ohad, N. , Margossian, L. , Hsu, Y.C. , Williams, C. , Repetti, P. and Fischer, R.L. (1996) A mutation that allows endosperm development without fertilization. Proc. Natl Acad. Sci. 93, 5319–5324. 11607683 10.1073/pnas.93.11.5319 PMC39243

[tpj12957-bib-0044] Ohto, M.A. , Floyd, S.K. , Fischer, R.L. , Goldberg, R.B. and Harada, J.J. (2009) Effects of *APETALA2* on embryo, endosperm, and seed coat development determine seed size in Arabidopsis. Sex. Plant Reprod. 22, 277–289. 20033449 10.1007/s00497-009-0116-1 PMC2796121

[tpj12957-bib-0045] Riefler, M. , Novak, O. , Strnad, M. and Schmulling, T. (2006) Arabidopsis cytokinin receptor mutants reveal functions in shoot growth, leaf senescence, seed size, germination, root development, and cytokinin metabolism. Plant Cell, 18, 40–54. 16361392 10.1105/tpc.105.037796 PMC1323483

[tpj12957-bib-0046] Rudella, A. , Friso, G. , Alonso, J.M. , Ecker, J.R. and van Wijk, K.J. (2006) Downregulation of ClpR2 leads to reduced accumulation of the ClpPRS protease complex and defects in chloroplast biogenesis in Arabidopsis. Plant Cell, 18, 1704–1721. 16766689 10.1105/tpc.106.042861 PMC1488914

[tpj12957-bib-0047] Sabelli, P.A. and Larkins, B.A. (2009) The contribution of cell cycle regulation to endosperm development. Sex. Plant Reprod. 22, 207–219. 20033442 10.1007/s00497-009-0105-4

[tpj12957-bib-0048] Schneider, C.A. , Rasband, W.S. and Eliceiri, K.W. (2012) NIH Image to ImageJ: 25 years of image analysis. Nat. Methods, 9, 671–675. 22930834 10.1038/nmeth.2089 PMC5554542

[tpj12957-bib-0049] Schruff, M.C. , Spielman, M. , Tiwari, S. , Adams, S. , Fenby, N. and Scott, R.J. (2006) The *AUXIN RESPONSE FACTOR 2* gene of Arabidopsis links auxin signalling, cell division, and the size of seeds and other organs. Development, 133, 251–261. 16339187 10.1242/dev.02194

[tpj12957-bib-0050] Sieberer, B.J. , Kieft, H. , Franssen‐Verheijen, T. , Emons, A.M. and Vos, J.W. (2009) Cell proliferation, cell shape, and microtubule and cellulose microfibril organization of tobacco BY‐2 cells are not altered by exposure to near weightlessness in space. Planta, 230, 1129–1140. 19756725 10.1007/s00425-009-1010-7 PMC2764053

[tpj12957-bib-0051] Sundaresan, V. (2005) Control of seed size in plants. Proc. Natl Acad. Sci. 102, 17887–17888. 16330781 10.1073/pnas.0509021102 PMC1312409

[tpj12957-bib-0052] Tanurdzic, M. , Vaughn, M.W. , Jiang, H. , Lee, T.J. , Slotkin, R.K. , Sosinski, B. , Thompson, W.F. , Doerge, R.W. and Martienssen, R.A. (2008) Epigenomic consequences of immortalized plant cell suspension culture. PLoS Biol. 6, 2880–2895. 19071958 10.1371/journal.pbio.0060302 PMC2596858

[tpj12957-bib-0053] Werner, T. , Motyka, V. , Laucou, V. , Smets, R. , Van Onckelen, H. and Schmulling, T. (2003) Cytokinin‐deficient transgenic Arabidopsis plants show multiple developmental alterations indicating opposite functions of cytokinins in the regulation of shoot and root meristem activity. Plant Cell, 15, 2532–2550. 14555694 10.1105/tpc.014928 PMC280559

[tpj12957-bib-0054] West, M. and Harada, J.J. (1993) Embryogenesis in higher plants: an overview. Plant Cell, 5, 1361–1369. 12271035 10.1105/tpc.5.10.1361 PMC160368

[tpj12957-bib-0055] Xia, T. , Li, N. , Dumenil, J. , Li, J. , Kamenski, A. , Bevan, M.W. , Gao, F. and Li, Y. (2013) The ubiquitin receptor DA1 interacts with the E3 ubiquitin ligase DA2 to regulate seed and organ size in Arabidopsis. Plant Cell, 25, 3347–3359. 24045020 10.1105/tpc.113.115063 PMC3809536

[tpj12957-bib-0056] Zhou, Y. , Zhang, X. , Kang, X. , Zhao, X. , Zhang, X. and Ni, M. (2009) *SHORT HYPOCOTYL UNDER BLUE1* associates with *MINISEED3* and *HAIKU2* promoters *in vivo* to regulate Arabidopsis seed development. Plant Cell, 21, 106–117. 19141706 10.1105/tpc.108.064972 PMC2648090

