# Supplementary figures and images for: Activation of CYCD7;1 in the central cell and early endosperm overcomes cell‐cycle arrest in the Arabidopsis female gametophyte, and promotes early endosperm and embryo development
Source: Plant J. 2015 Oct 1;84(1):41–55. doi: 10.1111/tpj.12957 (PMC5102630; doi:10.1111/tpj.12957)

Figure\_S1

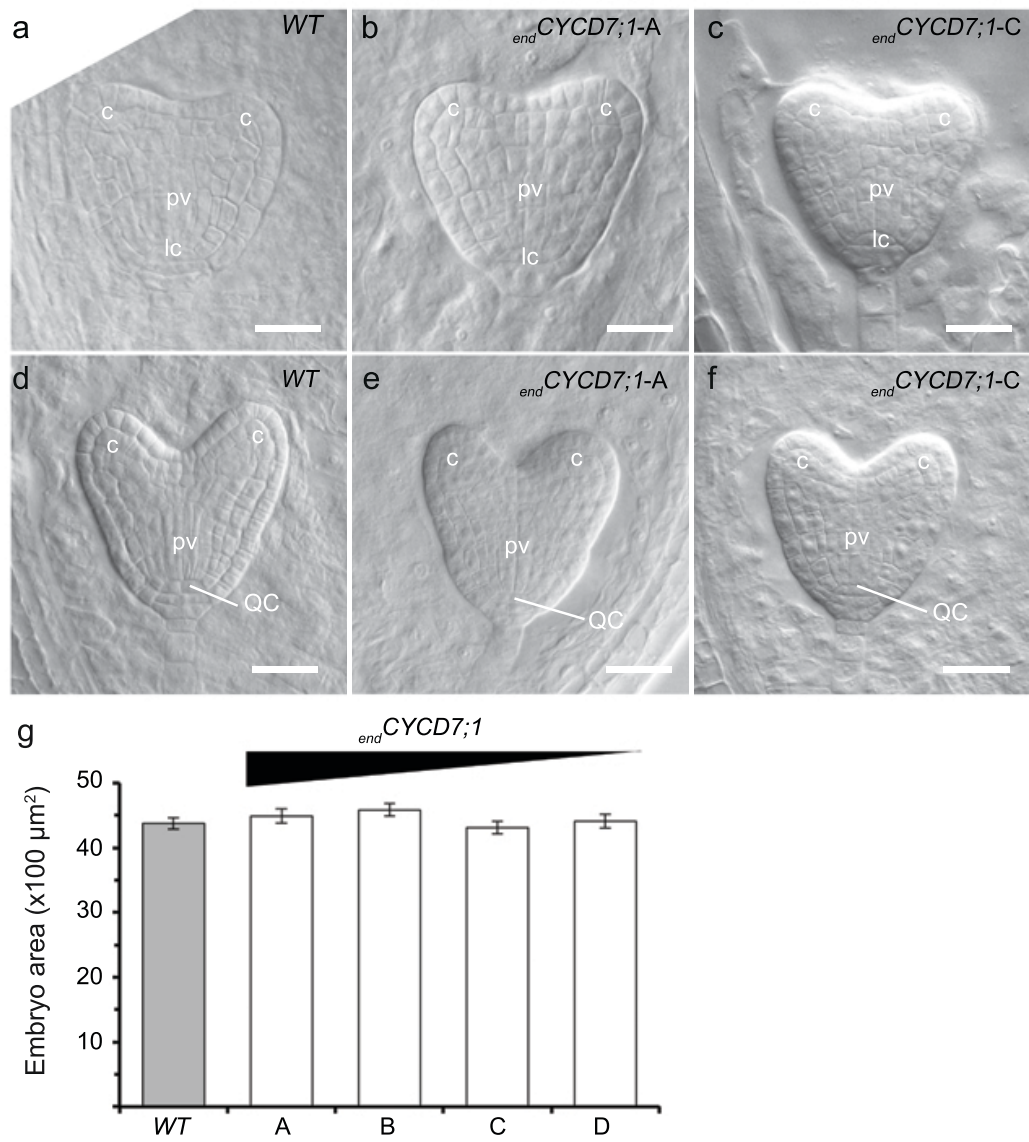

Supplement: Supplementary file 1 — Figure S1. Heart‐stage embryos in WT and end CYCD7;1 lines. [file TPJ-84-41-s001.pdf]

Figure\_S2

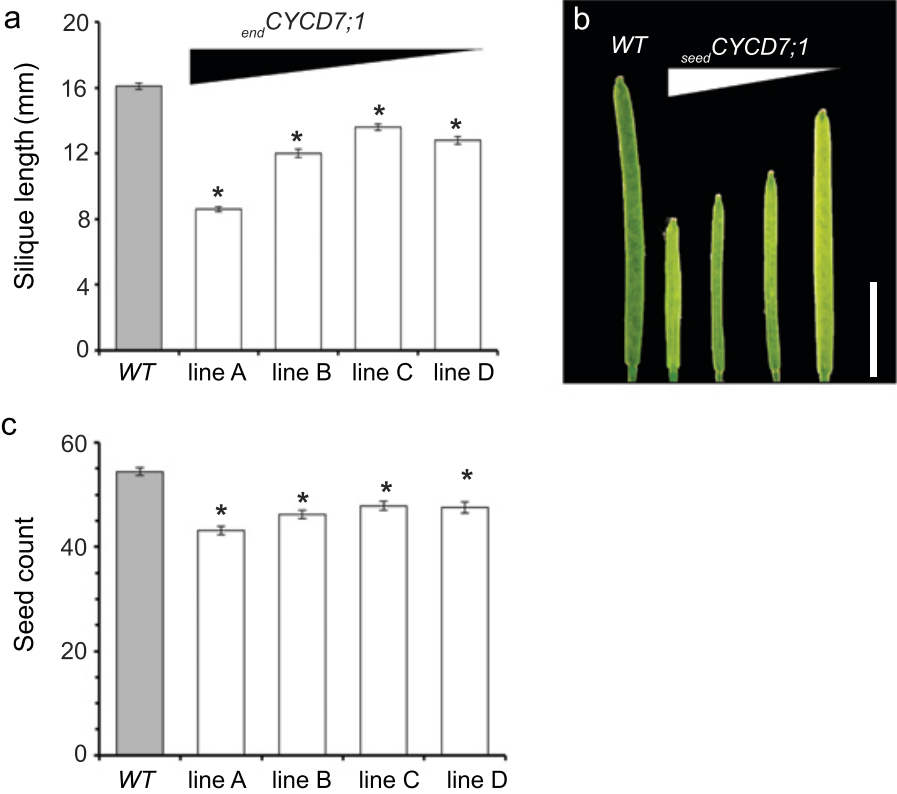

Supplement: Supplementary file 2 — Figure S2. CYCD7;1 expression under the activity of FWA induces an increase of seed abortion. [file TPJ-84-41-s002.R1]

Figure\_S3

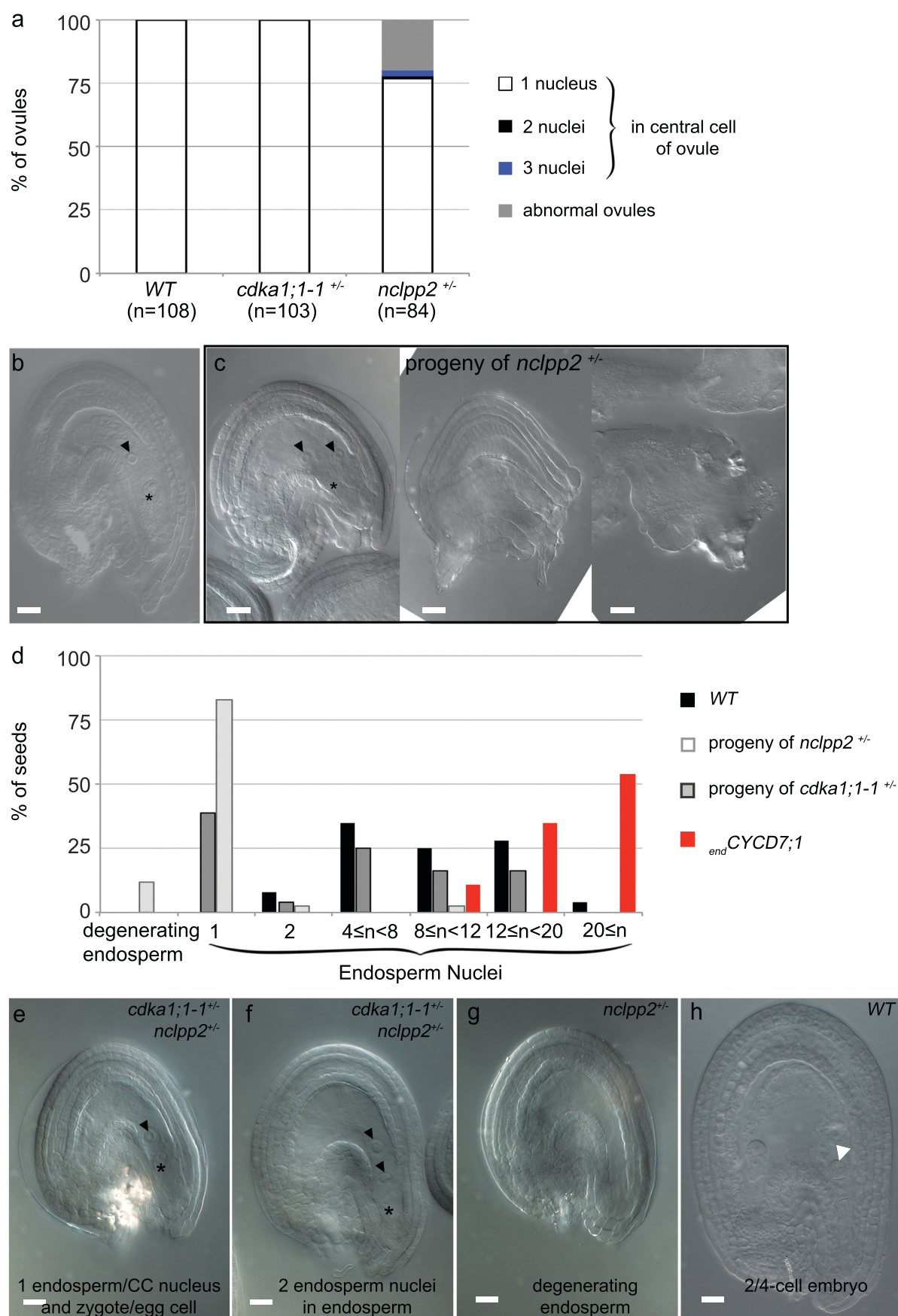

Supplement: Supplementary file 3 — Figure S3. Developmental characterization of cdka1;1–1 +/− and nclpp2 +/− mutant‐derived ovules and seeds. [file TPJ-84-41-s003.pdf]

Figure\_S4

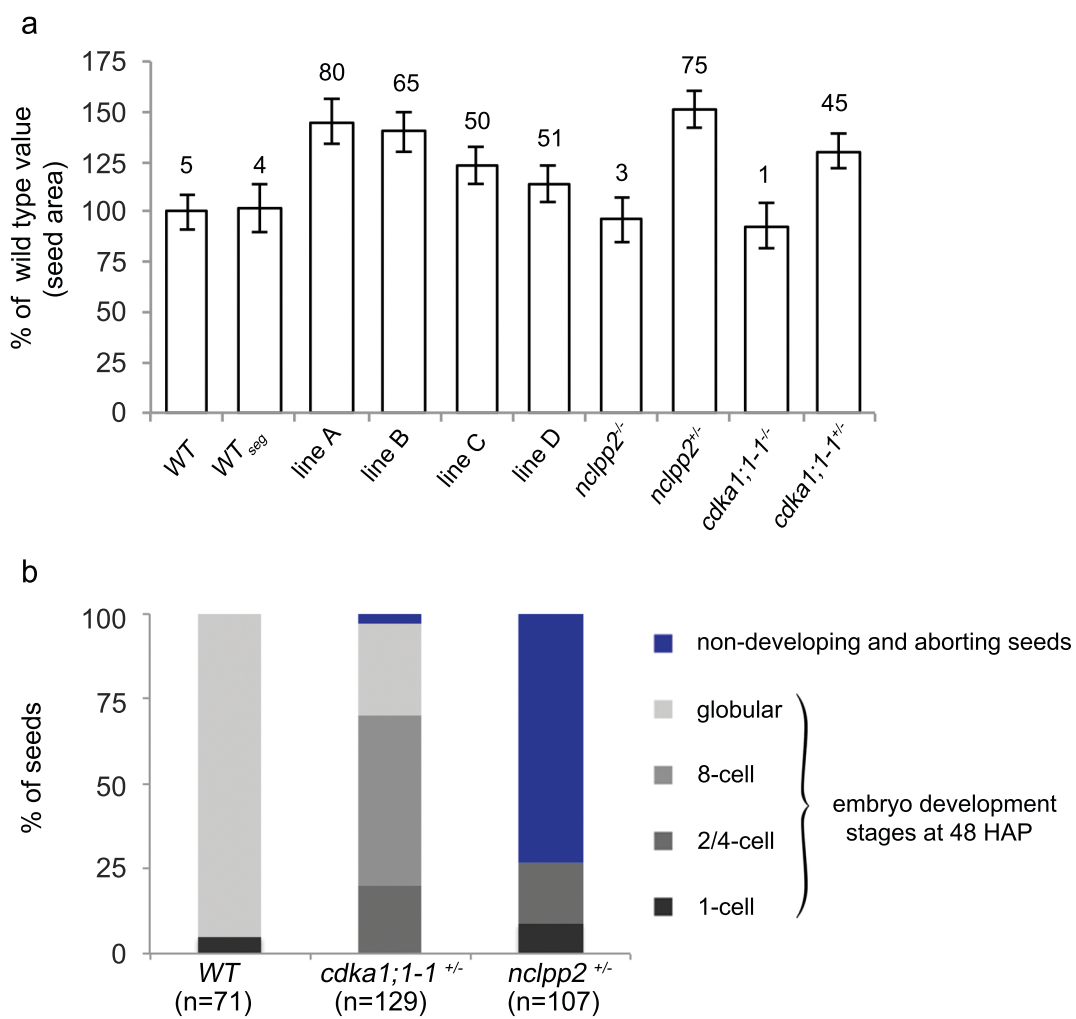

Supplement: Supplementary file 4 — Figure S4. Characteristics of mutant and end CYCD7;1 seeds. [file TPJ-84-41-s004.pdf]

Figure\_S5

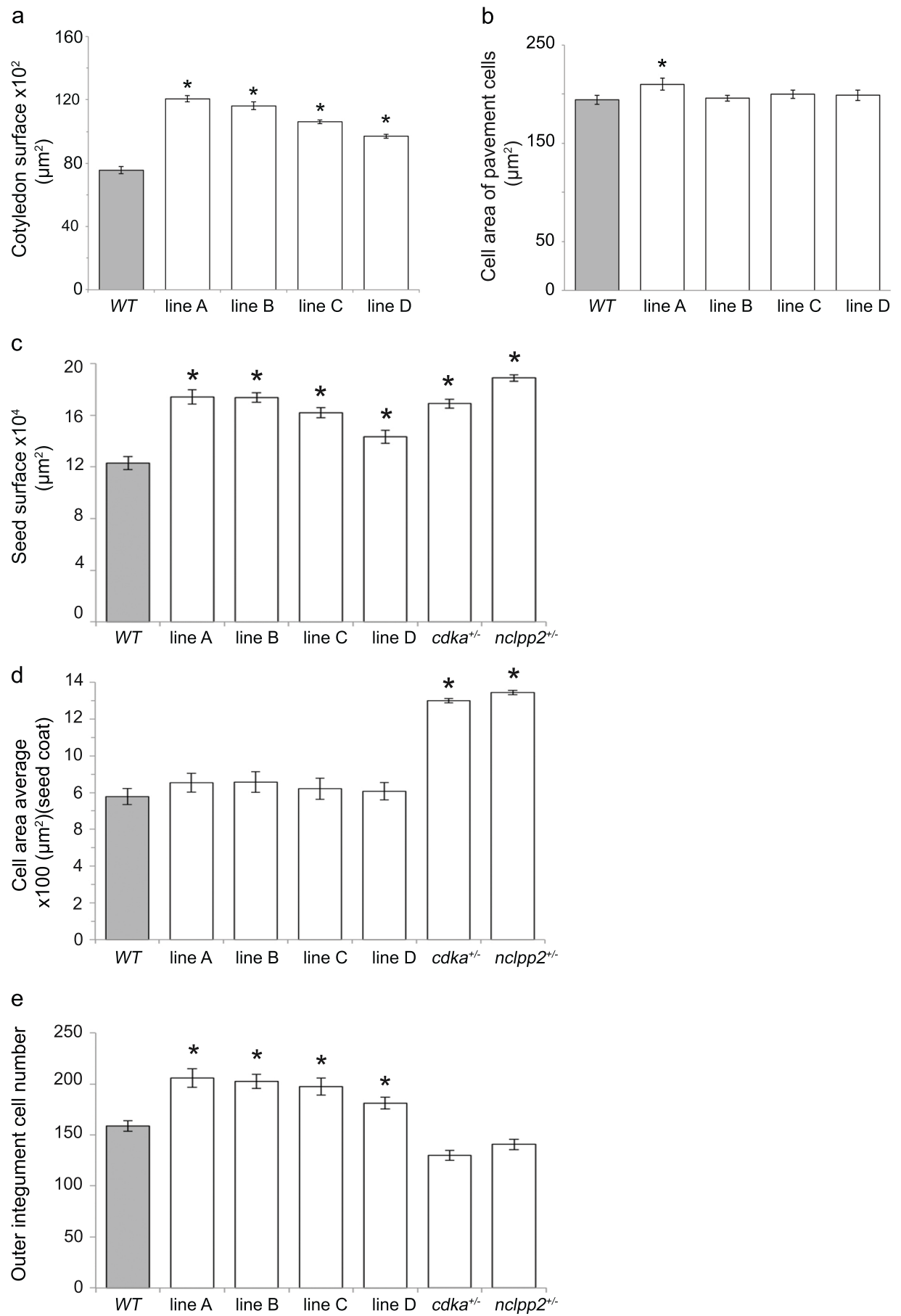

Supplement: Supplementary file 5 — Figure S5. Features of enlarged end CYCD7;1 mature seeds. [file TPJ-84-41-s005.pdf]

**CYCD7:1**

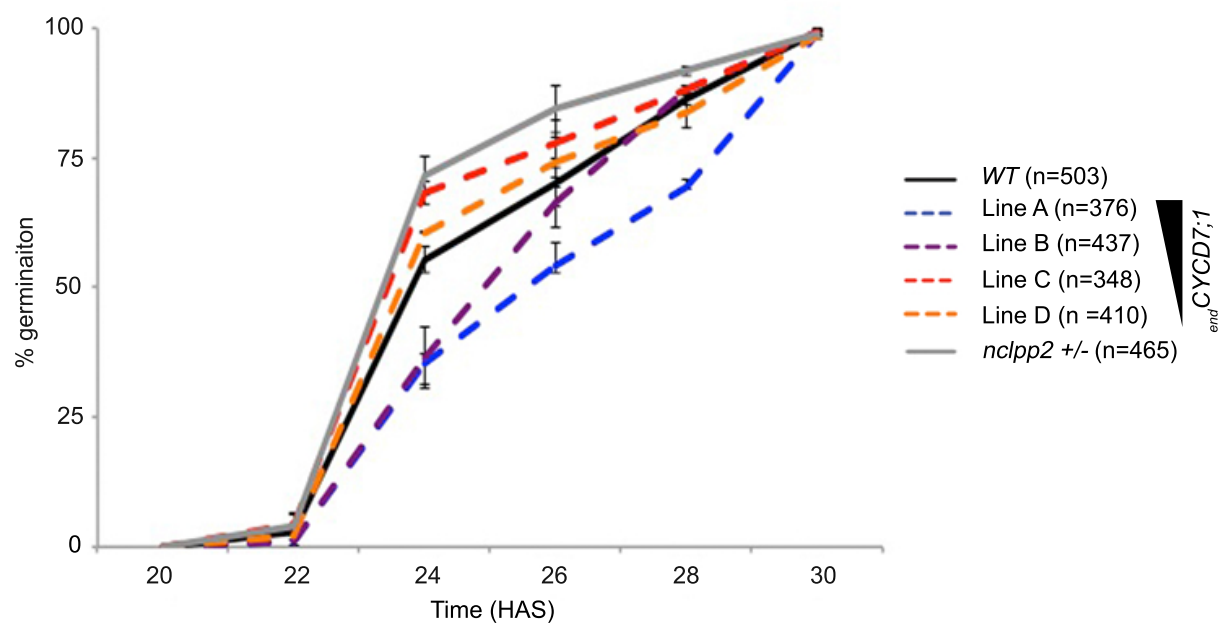

Supplement: Supplementary file 6 — Figure S6. Germination of seeds derived from end CYCD7;1, WT and nclpp2 +/−. [file TPJ-84-41-s006.pdf]
