# Supplementary material for: Activation of CYCD7;1 in the central cell and early endosperm overcomes cell‐cycle arrest in the Arabidopsis female gametophyte, and promotes early endosperm and embryo development
Source: Plant J. 2015 Oct 1;84(1):41–55. doi: 10.1111/tpj.12957 (PMC5102630; doi:10.1111/tpj.12957)
Supplement: Supplementary file 7 — Table S1. Reciprocal crosses between Col‐0 WT and end CYCD7;1 lines reveal a maternal origin of seed size increase (m, manual; sf, self‐pollinated). [file TPJ-84-41-s007.docx]

**Table S1.** Reciprocal crosses between *Col-0* *WT* and *_end_CYCD7;1* lines reveal a maternal-origin of seed size increase (sf, self-pollinated; m, manual).

| ♀ x ♂ | | | Pollination  procedure | Seed size ± SD  x10^3^ µm^2^ |
| --- | --- | --- | --- | --- |
| Col | x | Col | sf | 118 ± 8 |
| Col | x | Col | m | 102 ± 10 |
|  |  |  |  |  |
| A | x | A | sf | 158 ± 45 |
| A | x | A | m | 148 ± 24 |
| A | x | Col | m | 103 ± 27 |
|  |  |  |  |  |
| B | x | B | sf | 154 ± 26 |
| B | x | B | m | 143 ± 22 |
| B | x | Col | m | 103 ± 29 |
|  |  |  |  |  |
| C | x | C | sf | 149 ± 26 |
| C | x | C | m | 125 ± 21 |
| C | x | Col | m | 95 ± 27 |
|  |  |  |  |  |
| D | x | D | sf | 135 ± 27 |
| D | x | D | m | 116 ± 18 |
| D | x | Col | m | 90 ± 26 |
