# Supplementary material for: Activation of CYCD7;1 in the central cell and early endosperm overcomes cell‐cycle arrest in the Arabidopsis female gametophyte, and promotes early endosperm and embryo development
Source: Plant J. 2015 Oct 1;84(1):41–55. doi: 10.1111/tpj.12957 (PMC5102630; doi:10.1111/tpj.12957)
Supplement: Supplementary file 8 [file TPJ-84-41-s008.docx]

**SUPPORTING INFORMATION LEGENDS**

**Supplementary Figure S1. Heart-stage embryos in *WT* and *endCYCD7;1* lines.** (A-C) Early heart stage embryo and (D-F) late heart stage embryo from *WT* plants (A and D) and from *endCYCD7;1* lines (B and E line A, and C and F line C). Abbreviations: c, cotyledon; lc, lens-shaped cell; QC, quiescent centre; pv, provasculature. Scale bars: 15 μm. (G) Bar chart showing embryo area at heart- stage. Error bars show ± SE. No statistical difference between *WT* and *endCYCD7;1* is observed (one-way ANOVA, n>35, p=0.26). Relative expression of *endCYCD7;1* in the different lines is indicated by the triangle.

**Supplementary Figure S2. CYCD7;1 expression under the activity of the *FWA* promoter induces seed abortion.** Bar charts showing the length (A) and seed count (C) of mature siliques developing on the primary stem from position 10 to 14 form 15 different plants for each line; asterisk indicates a statistically difference when compared to *WT* (silique length: one-way ANOVA, n=75, p=3x10^-6^; seed count: one-way ANOVA, n=75 siliques p=2x10^-6^) and the relative expression of *endCYCD7;1* in the different lines is indicated by the triangle. (B) Picture of siliques at position 10 on the main stem of WT (first on the left) and *endCYCD7;1* plants (second from the left, line A, B ,C and D). Scale bar: 5 mm.

**Supplementary Figure S3. Developmental characterization of *cdka1;1-1^+/-^* and *nclpp2^+/-^* mutant derived ovules and seeds**. (A) Bar Chart showing the proportion of abnormal ovules and ovules with 1, 2 or 3 nuclei in the central cell of the female gametophyte in *WT, cdka1;1-1^+/-^* and *nclpp2^+/-^* emasculated pistils. (B-C) DIC micrographs of unfertilized ovules from *WT* (B) and *nclpp2^+/-^* (C: from left to right: examples of proper morphologically formed ovule however showing 2 nuclei in the central cell and ovules showing different degree of abnormality). (D) Number of nuclei in the seed endosperm 24H after pollination (43≤n≤56). (E-H) Early seed phenotypes in *WT,* *cdka1;1-1* and *nclpp2* mutants (24H after pollination). Black arrows indicate nuclei observed in the central cell of the female gametophyte. Star indicates the egg cell. White arrow points a 2/4-cell embryo. cc: central cell Scale bars: 20 µm.

**Supplementary Figure S4. Characteristics of mutant and *_end_CYCD7;1*** **seeds.** (A) Seed size in *WT* seed area of lines with ectopic activation of *CYCD7;1* in central cell and endosperm (*_end_CYCD7;1;* line A-D), and lines heterozygote for mutant alleles of *NCLPP2 (nclpp2^+/-^)*  and *CDKA (cdka1;1-1^+/-^)* including their respective *WT* controls *(nclpp2^-/-^; cdka1;1-1^-/-^)* (figures indicated the percentage lethality). (B) Developmental progression in seeds derived from *nclpp2^+/-^; cdka1;1-1 ^+/-^* plants 48H after pollination.

**Supplementary Figure S5. Features of enlarged *_end_CYCD7;1* mature seeds.** Cotyledon area (A) and cell area of pavement cells of cotyledons (B) of mature embryos of *WT* and *endCYCD7;1* lines. (C-E) Seed surface area and cell surface area of the outermost layer of integuments of the seed coat were analyzed using PI staining of cell walls and imaged by confocal microscopy. (C) Seed surface area. (D) Comparison of cell area in the outermost layer of the mature seed integuments. (E) Inferred numbers of cells in the outer integument by calculating the ratio seed surface:integument cell surface area. The asterisk indicates a statistically significant difference when compared to *WT.*

**Supplementary Figure S6. Germination of *_end_CYCD7;1*, *WT* and *nclpp2^+/-^* derived seeds.** Seeds were placed on a water-saturated filter paper in a closed environment. After 3 days of stratification at 4C in the dark, germination was scored at 21C with constant light. % germination is mean of 4 replicated of 82≤n≤138. Bars = SE. 50 % of all backgrounds germinate between 23 and 26 hours, and germination was completed after 30 hours.

**Supplementary Table S1. Reciprocal crosses between *Col-0* *WT* and *_end_CYCD7;1* lines reveal a maternal-origin of seed size increase** (sf, self-pollinated; m, manual).
